# Temporal coding enables hyperacuity in event-based vision

**DOI:** 10.1038/s41467-026-76878-6

**Published:** 2026-08-20

**Authors:** Eldad Assa, Alexander Rivkind, Michael Kreiserman, Fahad Shahbaz Khan, Salman Khan, Ehud Ahissar

**Affiliations:** 1 Weizmann Institute of Science, Department of Brain Sciences, Rehovot, Israel; 2 Mohamed bin Zayed University of Artificial Intelligence, Abu Dhabi, United Arab Emirates

**Keywords:** Retina, Neural encoding, Oculomotor system

## Abstract

Although the eye may appear static during fixation, it is in constant motion–transforming visual input into rich spatio-temporal streams of neuronal activity. These movements challenge the classical view that visual acuity derives from spatial sampling, suggesting that hyperacuity can emerge from precise temporal encoding. Here we show that artificial systems can exploit this principle using retina-like event-based (EB) sensing, a neuromorphic approach in which the sensor emits asynchronous spikes in response to luminance changes. Using an EB camera undergoing controlled “fixational” motion over tiny, pixelated images, we generated datasets in which recognition depends on sub-pixel information. We found that artificial neural networks trained on these spatio-temporal event streams relied crucially on the precise temporal information and outperformed conventional frame-based models. The learned representations also supported Vernier-style sub-pixel discrimination, demonstrating hyperacuity-like behavior in the artificial bio-mimetic system. These results show that active event-based sensing can use precise timing to recover spatial details that are unrecoverable from a static single frame. Together, these findings offer a new perspective on visual perception, open pathways for advancing neuromorphic engineering and energy-efficient AI vision systems, and provide a framework for testing hypotheses about biological vision.

## Abstract (web summary)

Unlike conventional frame-based vision, biomimetic event-based sensing preserves precise temporal information. The authors show that these timing signals can be exploited to recover spatial details from tiny images, enabling event-based vision to outperform frame-based approaches.

## Introduction

Human vision exhibits remarkable spatial precision, exemplified by visual hyperacuity, the ability to perceive fine details in small or distant objects with a precision exceeding the spatial resolution of retinal photoreceptors^1,2^. This is most famously demonstrated in Vernier acuity, where individuals can detect misalignments of only a few arcseconds, far smaller than the angular spacing of the smallest foveal cones^3^. Everyday tasks, from threading a needle to noticing slight crookedness or positional offsets, likewise draw on hyperacuity mechanisms that operate an order of magnitude beyond photoreceptor sampling^4^. Similar principles appear in other sensory systems, including tactile hyperacuity, where humans discriminate edge offsets finer than mechanoreceptor spacing^5^, and whisker-based object localization in rodents^6^.

During fixations, eyes drift at around 1 deg/s and amplitudes of several arcminutes^7^. Retinal cells respond to changes in illumination caused by the interaction between eye motion and local image contrasts during this fixational drift^7,8^. This form of active sensing enhances the perception of fine spatial details^9^. In a manner that may seem paradoxical, it also leads to a rapid loss of spatial correspondence between image details and retinal activations^10^, a phenomenon known as visual smearing^11^.

It has been hypothesized that the visual system may counteract smearing and achieve hyperacuity through spatial interpolation^1,12^, statistical inference^13^, backward suppression^14^ or by processing temporal delays between retinal spikes^8,15–17^ as the temporal component of spatio-temporal filters^18^. Evidence suggests that temporal differences can be perceived as spatial offsets^1,18,19^, and that humans achieve their highest performance when both spatial and temporal cues are present^20^. These observations suggest that temporal information may contribute substantially to high-acuity vision.

Additional evidence points to retinal mechanisms that transform spatial information into temporal signals: visual spatial resolution can be shaped by mechanisms inherited from the retina^21^, and luminance counterchanges at high-contrast borders can be translated into asynchronous neural activations^22^. This transformation of a spatial discontinuity into temporal patterns parallels the operating principle of EB sensors and raises a question relevant to both biological and artificial vision: can temporal information generated by active sampling help recover spatial details when the image is undersampled in space?

Tiny images constitute a particularly stringent test case in which spatial information alone is insufficient: the object occupies only a few sensor pixels, so fine spatial details required to recognize the object are eliminated by sensor pixelation. In this regime, even small temporal delays across neighboring pixels, induced by drift or motion, create discriminative cues that a static representation cannot capture^8^. Thus, tiny images expose the contribution of precise temporal encoding in a way that larger images, which already contain abundant spatial information, do not.

Event-based (EB) visual sensors generate events asynchronously, when individual photosensors detect luminance changes^23^, unlike frame-based (FB) cameras that collect synchronous information at fixed rates. Each such event contains precise timestamp, location, and polarity information. EB sensors have been used to model biological visual processing^24,25^, and their advantages have been demonstrated in many visual tasks (for a comprehensive survey of EB vision, see refs. ^26^, ^27^).

Although it has been shown that precise event timestamps contain information about the spatial structure of the image^28,29^, most EB image recognition models bin events over time, resulting in frame-like representations that discard this precise temporal information^26^. Furthermore, the ability to demonstrate the advantage of precise timing is limited by the fact that commonly used EB datasets for image recognition, such as^30^, contain sufficient spatial information^31^. As a consequence, most prior works incorporate temporal information into their representations without isolating its specific contribution^32–34^, and the potential advantage of precise temporal coding in EB sensing for image recognition has therefore only rarely been explored explicitly.

The current work aims to bridge this gap. Specifically, we show that using the temporal information embedded within visual EB data facilitates the recognition of tiny still images, such as digits or fashion items. Our experiments show that even images that appear unrecognizable due to pixelation by the photosensor array can be classified surprisingly well using bio-inspired visual signals that incorporate accurate temporal information, together with an appropriate classification model. We demonstrate this by showing that limiting the availability of temporal information degrades performance. We also collected corresponding FB datasets for comparison, and demonstrated the advantage of EB sensing in classification accuracy. Furthermore, the inherent relative motion and continuous acquisition in EB sensing allow us to exploit the constancy of the stimulus within each perceptual epoch to augment training and to train in an unsupervised, contrastive manner, demonstrating an additional advantage for EB coding.

Finally, by investigating our model’s internal representations, we show that training for image recognition in our challenging, near-threshold setting leads to the emergence of Vernier hyperacuity reminiscent of human performance. Additionally, we contribute a bio-inspired visual dataset that provides a benchmark in which fine temporal dynamics significantly affect image recognition performance.

## Results

### Creating tiny event-based versions of popular datasets

We used an EB camera (DAVIS240C, iniVation AG;^35^) to acquire visual events of static objects sampled through sensor motion. Using this camera, we converted three popular FB datasets (MNIST^36^, Fashion-MNIST^37^, and Kuzushiji-MNIST^38^) into EB datasets of tiny symbols. We acquired equivalent FB datasets for each condition (source dataset  × distance) in a separate session, using the same camera in FB mode (the DAVIS240C sensor produces both events and frames). The FB tiny-symbol datasets were collected with the camera stationary and the image centered on the silicon retina.

In total, we generated six EB datasets and six FB datasets, based on three source datasets and two distances. Their names combine the short name of the source dataset (MNIST, FMNIST, and KMNIST) with an ’ebt’ (EB tiny) or ’fbt’ (FB tiny) prefix, and a ’-D1’ or ’-D2’ suffix to indicate the camera’s distance from the display (e.g., the event-based dataset based on MNIST, generated with distance D2, is denoted ebtMNIST-D2). The ratio between pixel size on the sensor and on the display was estimated to be between 1:7 and 1:8 for D1, and between 1:12 and 1:13 for D2 (see Methods). The generated datasets are publicly available^39^.

**Fig. 1:**
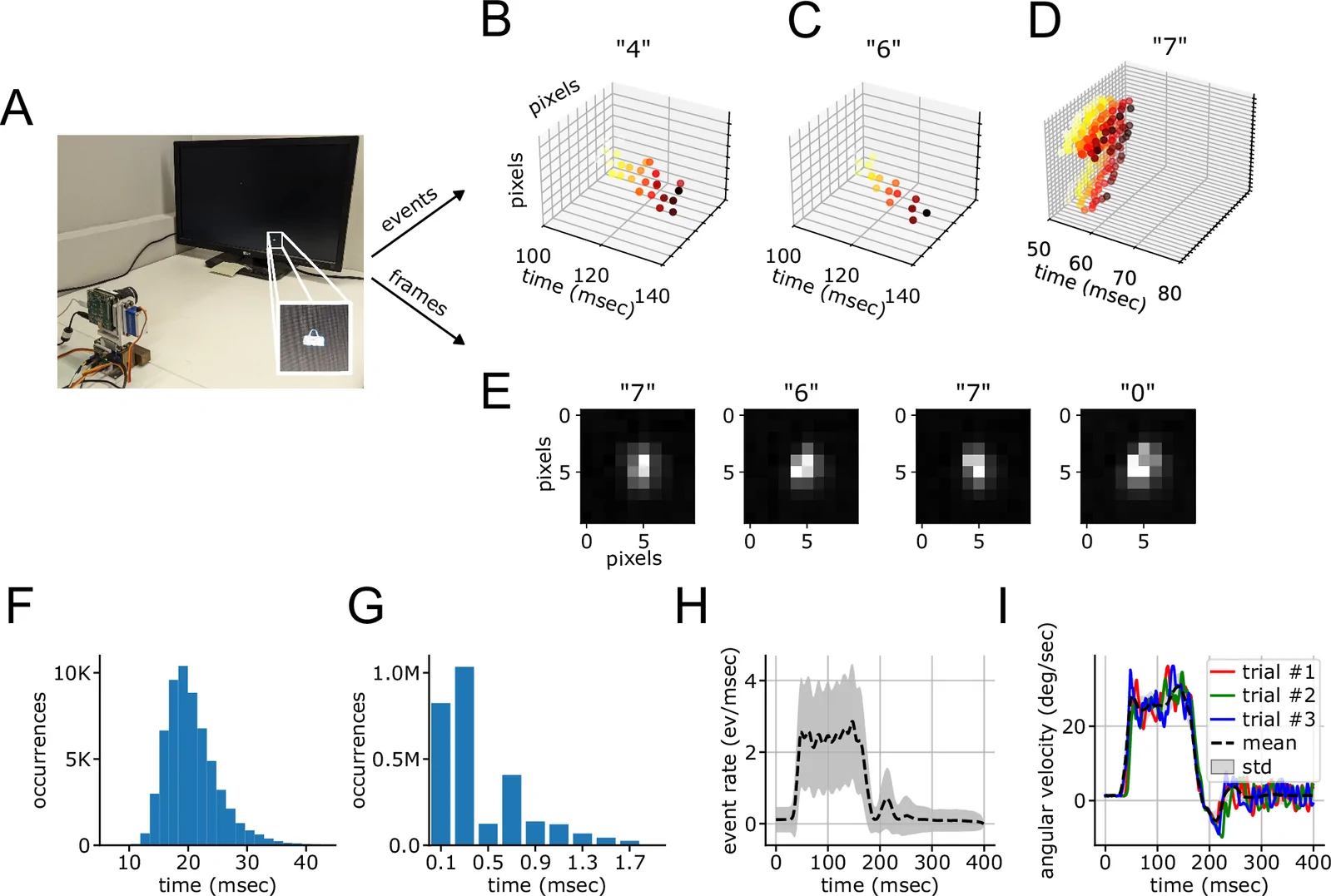
Experimental setup and data collected. **A** Photo of the setup used to acquire the tiny image datasets. The setup includes an EB camera mounted on a small robotic device, allowing it to rotate and a computer screen displaying the symbols. Symbols are displayed at their original resolution of 28 × 28 pixels, as shown in the inset in the bottom right corner. **B**, **C** 3D (*x*, *y*, *t*) raster plots of events acquired during a single motion while an MNIST symbol was displayed in front of the camera. The time axis is marked, and the orthogonal 2D plane represents the 2D sensor’s pixel matrix plane; point colors correspond to the events' timestamps (*t* value), with darker colors indicating later events. Only positive events are plotted. The label above each panel indicates the corresponding original symbol digit. **B** ebtMNIST-D1 sample; 28 events. **C** ebtMNIST-D2 sample; 18 events. **D** Full size, N-MNIST ^30^ sample; 180 events. **E** Cropped frames acquired with the event-based camera of four MNIST symbols at distance D1 (fbtMNIST-D1 dataset); the size of the cropped area is 10 × 10 pixels. **F** Histogram of the time it took the EB camera to generate 48 events, starting from t=100 ms (100 ms after the motor command was sent) for the 60,000 ebtMNIST-D1 dataset training set samples. **G** Inter-Event Interval (IEI) histogram. Histogram of the time differences between any two consecutive events over all the pixels on the sensor. **H** Event rate (events/ms) during a single horizontal rotation. Data from the ebtMNIST-D1 dataset acquisition (MNIST samples at distance D1). Mean  ± STD over 60,000 training set samples. **I** Horizontal angular velocity of the camera during the ebtMNIST-D1 dataset acquisition. The black dashed line and gray area represent the mean and STD over 60,000 training samples, respectively; colored traces (red, blue, and green) show traces from 3 single-sample acquisitions. **B**, **C**, **D**, **H**, **I** Time '0' corresponds to the motor command initiation. Source data are provided as a Source Data file.

Each dataset includes all 60,000 training samples and 10,000 test samples from the source datasets. The setup and examples of acquired events and frames are shown in Fig. 1A–C). As shown, the tiny symbols are hardly recognizable by the human eye when examining either the event streams (Fig. 1B, C) or individual frames (Fig. 1E). This is in contrast to event-streams from the pioneering work^30^, in which stimuli are large and are easily recognizable (Fig. 1D).

Under stable illumination, the camera generates events only if there is relative motion between the camera and the scene. Similar to^30^, we kept the scene static and moved the camera to create relative motion while avoiding artifacts from displaying motion on a digital display. We set the camera’s motion amplitude and velocity, and the size of the symbols on the sensor, as well as event rate, to approximately match the corresponding biological parameters (Supplementary Table S1).

### Information collected by event-based and frame-based sensing of tiny images

In order to assess the amount of information acquired in each sensing mode, we trained Artificial Neural Networks (ANN) models to classify the recorded samples. The performance of these trained models served as a benchmark for the information contained in the data. Thus, the models were not selected for their biological relevance but for their ability to decode information effectively.

To classify the EB data, we used a PointTransformer (PTr)^40^ classifier with  ~ 5.9M to  ~ 11.7M parameters (see Methods for implementation details). The model receives a time series of events (analogous to retinal ganglion cells’ spikes) presented as tuples of (*t*, *x*, *y*, *p*), where *t* is time, *x* and *y* are the spatial coordinates on the photosensor array, and *p* is polarity. This model was chosen mainly for two reasons. First, its a basic processing module is a Transformer^41^, which allows processing of sequences with long-range interactions between samples along the sequence. And second, this model provides access to temporal information with arbitrary precision, without temporal information loss. As a comparison, we also evaluated a gated recurrent unit (GRU) network^42^. As in the PTr model, the input was provided as tuples (*t*, *x*, *y*, *p*), so the GRU had access to the fine temporal information itself and not only to the ordering of samples in the sequence. The PTr performs slightly better than GRU;  ~ 5.6M parameters; Supplementary Fig. S1A).

The FB data were evaluated using ResNet-18 (11.7M parameters)^43^, which is a Convolutional Neural Network (CNN) architecture that gained remarkable popularity in conventional, frame-based, image processing.

Model’s hyperparameters (e.g., learning rate, number of layers and layers’ size) were determined through manual fine-tuning, supplemented by a partial grid search, using event-based MNIST with 48 events at distance D1. The only parameter adjusted for other datasets was the spatial centering (*x*, *y* coordinates). For the frame-based models, we used PyTorch’s built-in implementation of ResNet-18, with the frame-based input rescaled accordingly.

For more details on models and training procedures, see the Methods section below, as well as the public code repository containing the code used to define and train the models (see the link in Code availability).

**Fig. 2:**
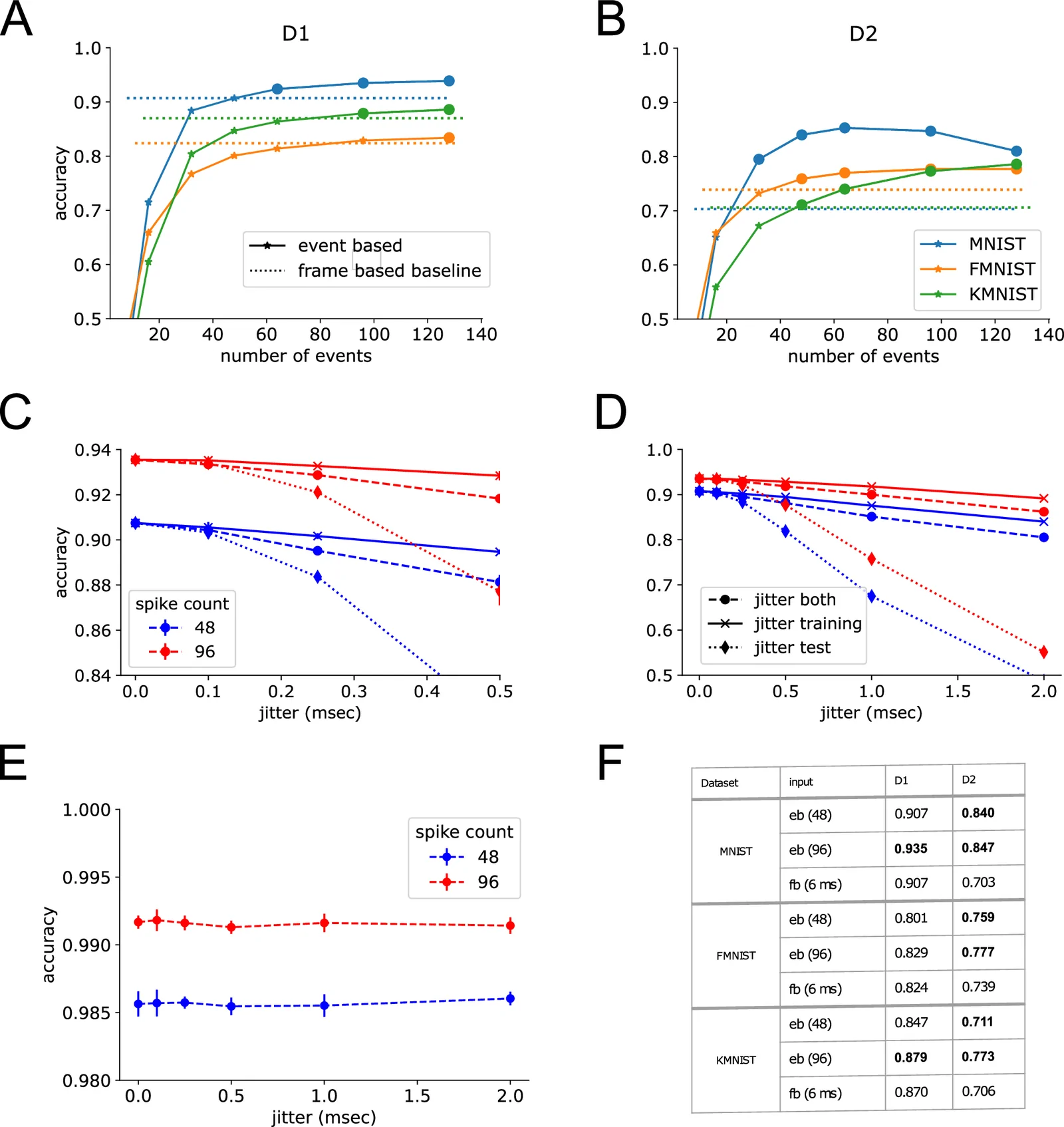
A, B Classification accuracy for tiny image datasets is shown versus the number of events, for acquisition distance D1 (A) and D2 (B). The dashed lines denote FB classification accuracy (Resnet-18) for the corresponding datasets and conditions. **C**, **D** Importance of temporal precision for tiny images: MNIST at distance D1 is evaluated in the presence of temporal jitter added during training, testing, or both. A zoomed-in version of the top left part of (**C**) is presented in (**D**). Different jitter conditions are indicated using different markers and line styles (see legend) and different event count is indicated using color (48 events- blue, 96 events- red). **E** Temporal jitter has no visible effect on classification accuracy for large images, as demonstrated on the N-MNIST dataset. Representative spatio-temporal raster plots are shown in Fig. 1D, and accuracy versus spike count is shown in Supplementary Fig. S1C. **F** Information from (**A**) and (**B**) presented in a table. Large circles in (**A**) and (**B**), as well as bold text in table in (**F**), correspond to conditions where EB models' accuracy is significantly different and larger than corresponding FB models accuracy (*p* < 0.05, two-tailed Wilcoxon rank-sum test over 5 training repetitions for each condition). In plots (**A**, **B**) mean accuracy is shown; in plots (**C–E**), Mean accuracy  ± STD is shown (as error-bars), in most cases error-bars are too small to be noticed (Supplementary tables Table S2 and Table S3 include the numerical values). Source data are provided as a Source Data file.

The accuracy of the PTr classifier increased monotonically and saturated rapidly as a function of the number of input events (Fig. 2A, B). For both D1 and D2 datasets, accuracy approached saturation within 50 to 100 events, with D2 datasets saturating more quickly (Fig. 2A, B). During camera motion, the mean event generation rate was approximately 2.5 events/ms for ebtMNIST-D1 (Fig. 1H) and 3.8 events/ms for ebtFMNIST-D1, while for ebtMNIST-D2 and ebtFMNIST-D2, the rates were around 1.5 and 2.1 events/ms, respectively.

For all conditions the accuracy of our model on EB data surpassed that of a standard CNN (ResNet-18) trained on the equivalent FB datasets within less than 128 events (Fig. 2A, B, F). For example, with ebtMNIST-D1, our baseline model (PTr, 5.8M parameters) started to exceed the accuracy of a standard CNN trained on fbtMNIST-D1 with about 48 events (Fig. 2A), eventually achieving a test-set accuracy of 0.924 ± 0.002 compared to 0.907 ± 0.001 for the FB network (Fig. 2A; *p* < 0.05, two-tailed Wilcoxon rank-sum test). ebtMNIST-D1 with 48 events was chosen as the default dataset and input size for all further experiments unless stated otherwise. Throughout the paper, we use a fixed number of events as the controlled parameter rather than a fixed time interval. However, for the basic setting (ebtMNIST-D1), we also report accuracy as a function of the time interval (Supplementary Fig. S1B).

**Fig. 3:**
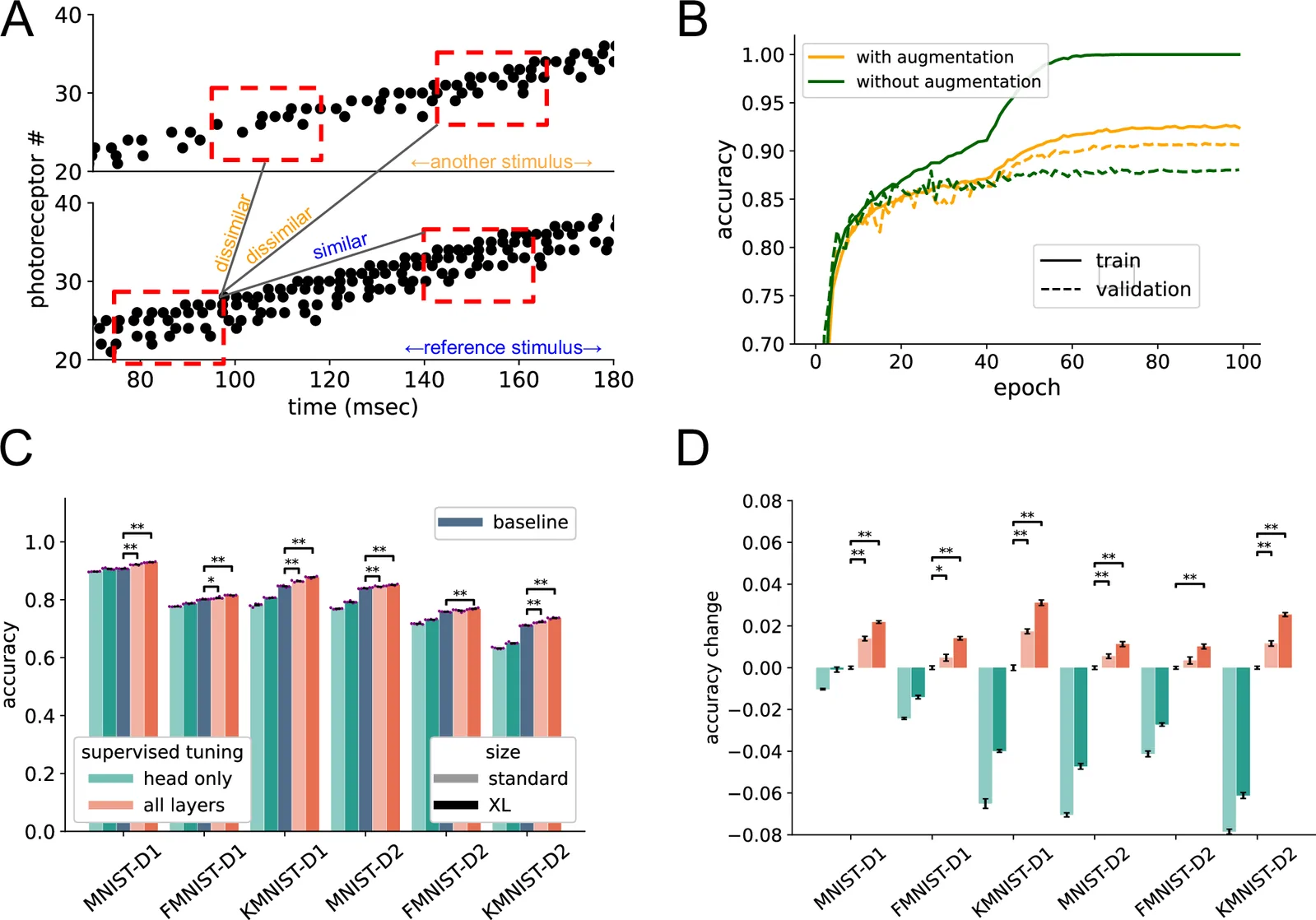
Data augmentation and contrastive learning with event sequences. **A** contrastive learning is achieved by rewarding the similarity of neural representations across different time intervals of the same stimulus while discouraging similarity when the intervals correspond to different stimuli. Augmentation is performed by using a variety of time intervals during the training phase. **B** Accuracy when learning from training data augmented by sequences from surrounding time intervals compared to accuracy when restricting training data to the test interval; train, train set; validation, validation set. Shown here is a representative learning curve for ebtMNIST-D1, 48 events.﻿ The learning rate slowdown began at epoch 40, which explains the abrupt rise in accuracy around this time. **C**, **D** The impact of contrastive pre-training is shown across datasets, network sizes, and fine-tuning methods. **C** Absolute test accuracies. **D** Differences relative to the reference classifier reported in Fig. 2 Small dots represent individual data. '*' and '**' denote *p* < 0.05 and *p* < 0.01 in two sided Wilcoxon ranksums test (no multiple comparisons were made). Precise *p*-values and Cohen’s effect size are supplied in the Source Data file.

A larger transformer model (~11.7*M* parameters, similar to ResNet-18) reached higher accuracy (0.929 ± 0.001 for the same event count) but required contrastive pre-training to converge (see Fig. 3C, Event streams are inherently augmented and suitable for self-supervised learning, and Methods).

### Accurate event timing contains task-relevant information

One of the main aims of this work was to illustrate the potential of using temporal information contained in event-based visual signals to decode spatial information. Assuming the trained EB ANNs learned to extract and use this temporal information to a high degree, degradation of this information at the network input would degrade decoding capability and reduce classification performance. To assess the significance of the sensor’s temporal accuracy for classification, we tested the transformer’s performance with artificial stochastic jitter added to the events’ timestamps. Added jitter values were sampled from a normal distribution with a mean of 0 and a standard deviation ranging from 0.1 ms to 2.0 ms (see Methods for details).

A clear degradation in classification accuracy was observed for the six EB datasets as the temporal noise level increased (Fig. 2C, D shows one representative dataset, and Supplementary Fig. S1 shows all datasets). This result indicates that the network learned to extract and use temporal information for the classification task. At a jitter magnitude of 0.1 ms, corresponding to  ~ 33% of the median inter-event interval (IEI, Fig. 1G; median is 0.33 ms), the impact of the added noise was negligible. However, increasing the noise to 0.25 ms ( ~ 75% of the median IEI) led to a significant degradation in classification accuracy (Fig. 2C; *p* = 0.02 for *jitter training* condition with 96 events, and *p* = 0.01 for all other combinations of jitter conditions (*jitter training, jitter both, jitter test*) and number of events (48, 96), two-tailed Wilcoxon rank-sum test).

Degradation of temporal accuracy could be applied to the samples used to train the model (the training set), to the samples used to test its final accuracy after training (the test set) or to both. The drop in final accuracy of the model compared to the no-added-jitter baseline for each of the three choices implies different things about the model’s reliance on precise temporal information. As evident from Fig. 2C, D, accuracy was degraded for all three conditions, and while the degradation of accuracy when testing with jitter and training without is not surprising, the two other observations are more interesting. First, applying jitter to both the training and test sets degraded accuracy, suggesting that the classifier failed to learn to overcome the noise. Next, we examined applying jitter only during training, while testing the model with unperturbed samples. Here, two opposing factors could theoretically play a role: jitter could augment the data by providing more training samples, but it could also compromise the model’s ability to capture fine-grained timing details, which are essential for accurate classification according to active sensing models^15^. Our results support the latter effect: even when jitter was added only to training data, while the test data remained intact, classification accuracy degraded. Thus, in our setting (unlike, e.g., ref. ^44^), the overall impact of training with jitter was negative; the degraded temporal information during training outweighed the positive effect that the jitter might have had in augmenting the training set.

On the other hand, loss of temporal precision can be compensated by a higher event count, or equivalently, a longer acquisition time. For example, for ebtMNIST-D1, the classification accuracy based on our default event number (48) with no added jitter ((0.907 ± 0.001; Fig. 2C) was approximately equivalent to processing 96 events with added temporal jitter of 1.0 ms (0.900 ± 0.003; Fig. 2C). Due to the high redundancy of information when representing  ~ 4 × 4 pixels symbol with 96 events and the stochastic nature of the added jitter, it might very well be the case that the 96-events inputs still contain significant amount of temporal information even with 2.0 ms STD jitter.

It has been shown that human observers can mitigate spatial noise by viewing for longer periods^45–48^. It would be interesting to experimentally test whether humans similarly mitigate temporal noise with longer fixations, using closed-loop gaze-contingent displays^49–51^.

However, we emphasize that while our results support the claim that temporal information plays a central role in classifying tiny images, it is redundant for full-resolution images, as demonstrated by ref. ^31^ for various datasets. We corroborated their finding using the N-MNIST event-based dataset^30^, which is based on full-resolution MNIST, by repeating the added jitter experiment with N-MNIST samples. This experiment showed no apparent effect from adding temporal jitter of the same magnitude that affected the classification of tiny images (Fig. 2E).

### Event streams are inherently augmented and suitable for self-supervised learning

Beyond providing precise temporal information, the unique properties of EB visual representations make them particularly well-suited for machine-learning approaches aimed at improving model performance. In particular, their active and dynamic sensing produces a rich, multifaceted signal that naturally supports data augmentation and self-supervised training.

Unlike FB systems, which frequently process identical frames repeatedly, EB systems are continuously exposed to a dynamic, ever-changing stimulus, making each encounter with the stimulus a truly “*Ichi-go ichi-e*" -" a unique, one-time experience. This inherent variability makes augmentation readily available and particularly effective. Here, leveraging the temporal nature of event sequences, we augmented the training data by varying the time windows from which event sequences were extracted. Specifically, during training, we used event sequences with starting times shifted randomly, uniformly within  ± 50 ms of the original time window used for testing. This method improved test accuracy by approximately 2.5% compared to using a non-augmented dataset (Fig. 3A, B). Because this augmentation arises directly from the intrinsic variability of EB sensing, it should benefit both biological and computer vision agents. Both can use it to learn to perceive challenging scenarios requiring rapid perception and where only a short glimpse of the scene is available.

Building on this inherent variability, we next turn to a self-supervised approach. Specifically, to further harness the benefits of EB data, we developed an unsupervised pre-training method using contrastive learning (e.g.,^52^ and references therein). The key idea was to obtain task-relevant neural representations in an unsupervised manner by enforcing temporal consistency: bringing similar (positive) pairs closer together in the network’s neural space while pushing dissimilar (negative) pairs further apart. We generated positive pairs by randomly selecting intervals from the same event sequence (we refer to these as time-based positive samples (TBPS)), and used intervals from different stimuli as negative pairs. This approach aligns with the idea, likely applicable to biological learning as well, that stimuli captured within short intervals (on the order of 200 ms) relate to the same external object (Fig. 3A). We trained our model following standard contrastive learning procedures with a projection head (see Methods for details) instead of the classification head (e.g., ref. ^53^), with positive pairs obtained via TBPS. After this pre-training, we replaced the projection head with a classification head and conducted supervised training, training either the entire model or just the classification head, to optimize its performance as a classifier.

We evaluated the standard outcomes of contrastive training in our TBPS setting, focusing on three key aspects: (i) the amount of information contained in the resulting neural representation, determined by its ability to support a downstream task (classification, in our case) by adding a simple decoding head; we quantified this by comparing its performance to that of a fully supervised model; (ii) the extent to which TBPS-based contrastive pre-training improved performance when the entire model was fine-tuned; and (iii) whether TBPS-based contrastive pre-training enables the training of larger models that would otherwise fail to converge. Fig. 3C, D presents the outcomes of TBPS-based contrastive pre-training across various datasets. The x-axis shows the baseline reference model (middle), with results displayed for TBPS-based contrastive pre-training followed by fine-tuning of the head alone (left) and full fine-tuning (right). In the head-only fine-tuning scenario, where only the classification head was trained while the rest of the model remained frozen, the results were only slightly inferior to those achieved with full fine-tuning (addressing question (i)), demonstrating that temporal contrastive pre-training produces representations nearly sufficient for downstream classification tasks. TBPS-based contrastive pre-training further significantly improved performance when the entire model was fine-tuned (addressing question (ii)), confirming its utility in enhancing model capabilities. Furthermore, we evaluated an enlarged model, denoted ’XL,’ with a parameter count close to that of ResNet-18 (11.7 M), previously used as an FB classifier in Fig. 2A, B. TBPS-based contrastive pre-training proved essential for the XL model, as it failed to converge under vanilla supervised training (addressing question (iii)). These results highlight the dual advantages of TBPS-based contrastive pre-training in constructing effective representations and enabling the training of larger architectures.

### Testing for Vernier hyperacuity

Humans can resolve Vernier offsets with *hyperacuity*^1,2^; that is, they can distinguish offsets that are remarkably small relative to a photoreceptor’s receptive-field size. Does the ability to recognize tiny objects through temporal coding generalize to this standard hyperacuity task?

**Fig. 4:**
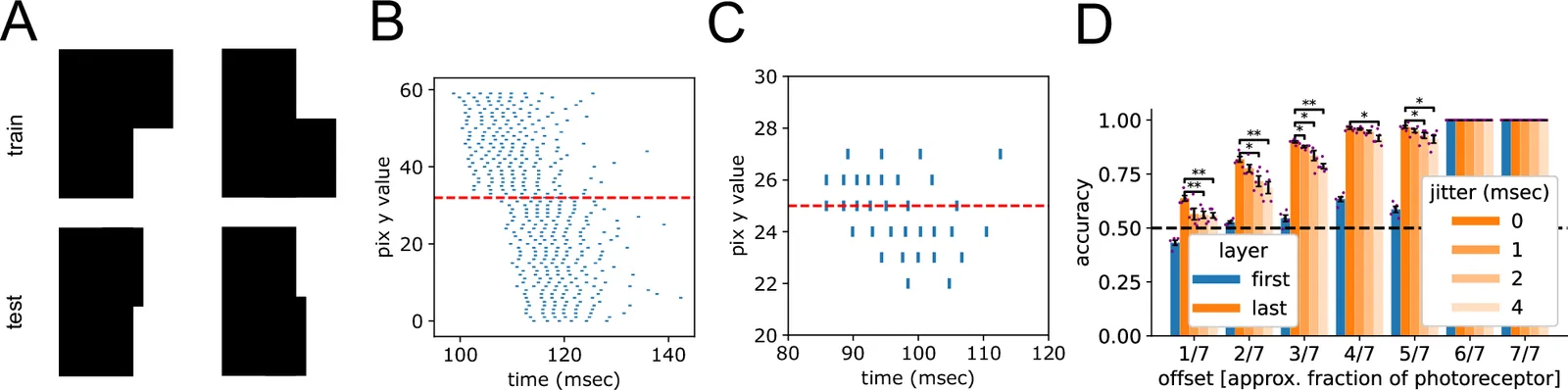
Vernier task. A Task illustration: The agent must report whether the top of the bar is shifted left or right relative to the bottom. **A** Training is done with large offsets, and testing is performed with small offsets. **B** A sensor activation raster plot showing events emitted by a column of 60 photosensors when crossing a Vernier stimulus like the one shown in (**A**; top-left) while rotating to the right. Numbers on the y-axis correspond to the photosensor index within the column. The dashed red line indicates the vertical position of the Vernier bar shift. Events below and above the red dashed line are shifted by less than 10 ms, corresponding to a sensor speed of  ~ 30 deg/s. Events are not perfectly aligned in time across photosensors due to sensory and motor noise; a single crossing of the edge results in the generation of bursts of events lasting  ~ 15 ms. **C** Same as (**B**) but for a small edge where the edge’s vertical dimension on the display is equivalent to a single MNIST symbol (28 pixels), corresponding to  ~ 5 photosensors at D1 distance. The examples depicted in (**B**) and (**C**) correspond to an offset of a single photosensor (large offset) for clarity. **D** Accuracy in the Vernier task with small edges (see **C**). Results are shown for readout from the first and last transformer block in an ANN trained as a classifier of ebtMNIST-D1. Offsets of 6-7 pixels were used as training data, and offsets of 5-1 pixels were used as test data. Between 50 and 51 examples of each offset were presented. Error bars indicate Mean  ± SEM values. Shades of orange represent the added jitter (shown for the last layer only). Black dashed line indicates the chance-level accuracy. Small dots represent individual data. '*' and '**' denote *p* < 0.05 and *p* < 0.01 in two-sided Wilcoxon ranksums test (no multiple comparisons were made). Precise *p*-values and Cohen’s effect size are supplied in the Source Data file.

To address this question, we enriched the ebt-MNIST dataset with Vernier-like stimuli featuring offsets ranging from 1 to 7 screen pixels in both directions (Fig. 4A–C). These stimuli were presented interleaved with the MNIST data during acquisition to ensure that mechanical parameters (e.g., sensor operating temperature) were consistent across datasets. To better reflect naturalistic scenarios, we used the single-edge version of the Vernier task^54^, rather than the more commonly used version with isolated bars or lines.

To emulate a common psychophysical setting, in which observers, already familiar with everyday objects, are tested with Vernier offsets, we used a model pretrained on ebt-MNIST and tasked it with Vernier offsets. To further mimic psychophysical experiments, we first trained a linear classifier on the model’s internal representations using large Vernier offsets of 6-7 monitor pixels (corresponding to $\frac{6}{7}$ to $\frac{7}{7}$ of photosensor size), which do not require hyperacuity. We refer to this classifier as the Vernier-classifier. We then tested this classifier on smaller offsets down to a single screen pixel (approximately $\frac{1}{7}$ of a photosensor).

We found that the Vernier-classifier performed well when trained on the output of the last layer of the base model, but not when trained on the output of the first layer (Fig. 4D). This indicates that the model’s internal representations are sufficiently rich to generalize from digit recognition to the Vernier task. To assess whether performance depended on the temporal differences between events, we measured accuracy while applying temporal jitter to the input. Performance degraded significantly at a jitter of 2 ms (except for the $\frac{4}{7}$ offset, for which degradation reached significance at a jitter of 4 ms) (Fig. 4D). This is consistent with the qualitative visualization in Fig. 4B, C, which shows how a spatial offset is translated into a temporal offset between events. We note that the effects on accuracy are expected to be more moderate in the Vernier setting, which is a two-alternative task, compared with digit classification, which is a ten-alternative task.

Whether biological Vernier hyperacuity relies primarily on spatial or spatio-temporal information has been debated^1,8,20^. While our current results provide a proof of feasibility for the temporal hypothesis^8^, they cannot decisively help resolving this debate; the raw Vernier stimulus in our FB condition, which does not include eye movements that are inherent to the biological system, was sufficient for accurate decoding of all Vernier offsets (Supplementary Fig. S3).

## Discussion

When classifying tiny objects, purely spatial information is limited by the resolution of the photosensor array (Fig. 1B, C) and further degraded by sensor motion. We showed that using spatio-temporal rather than purely spatial coding turns sensor motion into a feature rather than a bug^6,8,55^, thereby enabling recognition of spatially undersampled images. These results highlight the value of temporal precision for event-based vision. They also motivate further experimental investigations of how temporal signals generated by eye movements may contribute to biological visual acuity, extending beyond the specific Vernier task demonstrated here (Fig. 4).

Our results, obtained using a robotic bio-inspired vision system, align with the view that biological visual processing relies on both temporal and spatial information^7,15,56^. Specifically, we demonstrated that longer viewing times improve classification accuracy (Fig. 2), consistent with human studies^45–48^. Furthermore, we replicated a standard hyperacuity experiment (Fig. 4), showing that training on tiny images is sufficient to resolve Vernier stimuli with human-like hyperacuity^57^. We also demonstrated the benefits of continuous scene sampling for training. When combined with the assumption that high-level perceptual representations remain stable over time, such sampling enables unsupervised training (Fig. 3). Finally, we benchmarked the classification accuracy of EB and FB systems and found that EB performance was comparable or superior for distant, small images. Moreover, the advantage of EB became more pronounced as viewing distance increased (Fig. 2A, B).

At the same time, it is important to clarify the scope of our conclusions. Our study addresses sensory encoding only; we do not model higher-level perceptual inference or cortical learning. Our experiments were designed to explore a specific component of the sensory mechanism: the contribution of temporal precision in spatially undersampled input signals. We acknowledge that the biological visual system likely employs a variety of mechanisms, including spatial, spatiotemporal and asymmetric polarity-based mechanisms^21,22^ to achieve accurate perception.

Temporal information from EB vision has been shown to contribute to perceiving dynamic scenarios such as action recognition^31^, optical-flow computation^25,58^, and 3D reconstruction from stereo-vision^59,60^. Temporal precision was also shown to be beneficial for overcoming smearing through motion compensation or cancellation^61,62^. More generally, prior work has demonstrated that spatio-temporal information can contribute to recognition performance in both frame-based and event-based systems^32,55,63^.

However, despite demonstrations that precise timestamps of visual events generated from static images contain information about the presented visual pattern^28^, EB cameras are commonly considered less suitable for perceiving static scenes^26^. This is primarily because the EB signal is generated by relative motion; thus, for static scenes, the camera must move, resulting in a distorted representation of the scene. Indeed, for the relatively few cases where large EB static image datasets had been used for image recognition^34,64–68^, the datasets being used (N-MNIST, N-Caltech101^30^) were shown not to contain additional discriminatory information in the time domain^31^. This was done by showing that training a CNN on frames generated by “time-collapsing" EB data results in equivalent or superior performance compared to EB spatio-temporal models^31^. This was confirmed also by ref. ^34^ where they utilized temporal jitter experiments to assess the robustness of their model to temporal noise, and found that for N-MNIST, jitter with a standard deviation as large as 10 ms did not affect the model’s accuracy even in the most challenging setting where jitter is applied only to the test set.

Here, we focused on a regime in which spatial information alone is insufficient. By considering tiny images, we isolate conditions under which temporal coding becomes critical for successful recognition, rather than merely advantageous. Accordingly, our results challenge the general assumption described above and differ from those showing that fine temporal resolution mainly contributes to precise motion compensation^26^, for example, by “canceling" motion and generating static representations of a scene^13^. In our case (Figures 2–4), precise temporal information is used to construct representations rather than to compensate for perturbations, achieving superior performance compared to equivalent spatial frame-based models.

The model we used, a PointTransformer^40^, is not biomimetic. Rather, we leveraged its ability to process sequences of spatio-temporal tuples, preserving precise temporal information when handling event streams, to assess the value of precise temporal information for image recognition (Fig. 2). Future work aimed at understanding biological vision could extend these findings to more biomimetic models, including asynchronous networks (SNNs) and biologically inspired learning rules, implemented on neuromorphic hardware^69–71^, while generalizing to more naturalistic input scenarios.

Another central direction for future work is the development of a more biologically plausible curriculum of learning: humans and animals may learn to perceive high-resolution views before generalizing to degraded inputs, whereas our networks were trained directly on undersampled images. This design emphasizes the role of temporal coding under conditions of minimal spatial information. Importantly, however, the present work shows that ANNs using EB data as an input can learn useful representations in an unsupervised manner (Fig. 3C), based solely on the temporal constancy of the input. This suggests that, in the more general case where recognition is not restricted to tiny images, generalization from typical images to very small ones may be supported by such learned representations. In other words, even if the learner is not routinely exposed to meaningful tiny images, the feature space required to process them may still emerge through unsupervised learning and be recruited when needed. Future work combining high- and low-resolution training could more closely capture biological learning dynamics.

With spatio-temporal coding, the resolution of encoding external space is determined by a combination of spatial and temporal factors. The spatial limiting factors are the fixed geometry of the photosensor array and its optics. The temporal limiting factors are the temporal precision of the photosensors and the sensor motion speed (see Fig. 4C). When exploiting the temporal coding domain, biological agents can adjust sensor speed to balance increased sensing resolution (achieved at lower speeds) against reduced overall recognition time (achieved at higher speeds)^51,72^. In our ebtMNIST-D1 experiment, our default event count of 48 events was typically acquired over a time interval of approximately 20 ms (Fig. 1f) with horizontal velocities of  ~ 30 deg/s (Fig. 1I). Thus, symbols were typically shifted by more than 2 pixels on the sensor during the acquisition of the default event count, corresponding to  ~ 70 − 80% of the tiny symbol size. In head-fixed conditions, human ocular drifts exhibit instantaneous speeds on the order of 1 deg/s^7^. Assuming foveal receptive fields are on the order of 0.01 deg in diameter^73^, an edge moving across neighboring receptors would generate temporal delays on the order of 10 ms. Assuming a neuronal temporal resolution of approximately 1 ms^16,74^, spatial offsets as small as one tenth of a photoreceptor’s diameter could, in principle, be resolved. Such sensitivity is demonstrated in our bio-inspired robotic vision system (Fig. 4). Furthermore, the importance of high temporal resolution is evident from our findings on tiny-image classification results, which show that temporal jitter as small as 0.25 ms significantly reduces accuracy (Fig. 2C, D).

The above comparison (as well as the comparison in Supplementary Table S1) focuses on statistical measures which involve motor-sensory retinal parameters and their interactions, and demonstrate the potential biological relevance of our results. They do not, however, rule out alternative biological mechanisms. Hence, further validation of such a biological relevance should include comparison to results achieved in human experiments, which would require adding missing biological degrees of freedom to our setup.

Recent experiments indicate that ocular drift is not fully stochastic; its speed and curvature adapt to the visual stimulus^50,51^. Our data, collected in an open-loop, stimulus-independent manner, can help design closed-loop visual systems. In these systems, camera motion is part of the perceiver-environment dynamics, where motion optimizes sensory and perceptual efficiency. In our setting, motion and sensory parameter values partially overlap with those typically used by human observers as mentioned above (Supplementary Table S1). The advantages and limitations of human-like parameter regimes can be tested in future biomimetic applications of closed-loop vision.

Our work also shows how an event-based representation enables unsupervised, contrastive learning. This approach leverages the inherent variability of subsequences within an event sequence related to a static object (Fig. 3A) to facilitate learning (Fig. 3C, D). Specifically, our paradigm used different spike trains, generated during a single symbol acquisition, to train internal representations of the same category (Fig. 3). It is reasonable to expect that, similarly to our paradigm, the brain uses prior knowledge of the approximate constancy of the environment over tens of milliseconds to develop useful representations. Future work could extend this unsupervised approach to train a generic EB model without requiring extensive labeled datasets.

Engineering models inspired by biology have often become useful testbeds for neuroscience, even when the biological circuitry is not literally emulated. Convolutional neural networks, for example, have enabled systematic comparisons between task-optimized artificial representations and the primate ventral visual stream (e.g., ref. ^75^), while recurrent neural networks trained on cognitive tasks have helped generate and test hypotheses about cortical population dynamics (e.g., ref. ^76^). We hope that the datasets introduced here will serve an analogous role for temporally precise, event-based representations. By isolating settings in which fine event timing matters, these datasets provide a controlled testbed for studying spike-level codes, active sampling, and downstream decoding in artificial systems, and for comparing the resulting representations with neural or behavioral measurements in biological vision. Such comparisons can sharpen biologically motivated hypotheses without requiring the artificial models to be treated as mechanistic models of the brain.

In conclusion, we revealed two previously unappreciated advantages of bio-inspired EB sensing: its superior ability to perceive tiny images through precise temporal coding and its effectiveness in training. While these advantages were demonstrated here on relatively simple datasets, the findings motivate extending the approach to more complex real-world visual tasks and to more biologically constrained models. They also suggest that similar mechanisms may contribute to human vision, a hypothesis that remains to be tested directly. Finally, we provide controlled datasets that expose temporal-coding effects difficult to isolate in standard benchmarks, offering a resource for event-based vision research and a platform for testing biologically motivated hypotheses about active visual sensing.

## Methods

### Setup

We used an EB camera (DAVIS240C, iniVation AG;^35^) to generate event streams of small static objects while rotating. To allow controlled rotations, the camera was mounted on a pan-tilt unit (PTU) composed of 2 servo motors (TGY-D003HV, TURNIGY), a small motion controller (Mini Maestro, Pololu), and mechanical parts produced in-house. A computer display was positioned in front of the camera (Fig. 1A). This setup was inspired by ref. ^30^.

The camera, controller, and display were connected to a single computer that saved the camera’s output (events/frames, Inertial Measurement Unit (IMU)), initiated and ended the camera’s cyclic rotation, and displayed the symbols. The camera’s IMU continuously recorded the camera’s angular velocity at a sampling rate of 1 kHz (Fig. 1I). One of the controller’s digital outputs was connected to the camera’s trigger-in input to accurately synchronize the start and end of rotation movements with the visual events generated by the camera.

Python code coordinated the display of symbols, the camera’s motion, and the acquisition of files containing the camera’s output. We used the dv-runtime software (iniVation AG; version 1.6.2) to initialize the camera, set its parameters, and retrieve its output. The camera’s data was saved to files on the computer using this framework.

### Datasets acquisition

We used our setup to convert popular image classification datasets into EB and FB tiny image datasets. The source datasets’ symbols were projected onto the computer display at their original resolution of 28 × 28 pixels (Fig. 1A, inset). We recorded datasets with the camera at one of two distances from the display: 525 mm (configuration ’D1’) and 830 mm (configuration ’D2’). In configuration ’D1,’ the symbol covered  ~ 5 × 5 sensor pixels, while in ’D2,’ the projected symbol size was  ~ 4 × 4 pixels (see below for details on projection size).

The datasets were acquired using simple motion profiles involving either a single fixed-velocity horizontal rotation (pan) or a fixed-velocity pan followed by a fixed-velocity vertical rotation (tilt). While the nominal speed was fixed, the actual speed varied significantly, as shown in Fig. 1I.

Illumination conditions were kept constant during all dataset acquisition sessions. The setup was placed inside a dark room, which resulted in a consistently dim environment, as the computer display was the primary light source reaching the camera. The display background was set to 20% brightness, and stimulus pixel values (0-255) were linearly mapped to the 20%-100% gray level range.

The camera’s biases were also kept constant for all acquisition sessions (the dv-runtime configuration file, including camera biases, is included in the GitHub repository). For FB datasets, the camera’s frame rate was set to 20 Hz, with an exposure time of 6 ms. The exposure time value was chosen by acquiring fbtMNIST-D1 datasets with different exposure times and choosing the one with which the model reached maximal performance (See Supp Fig. S2). The same exposure value was then used for all other FB datasets.

The dv-runtime initially saved acquired data in aedat4 format. Pre-processing, before training the networks, involved parsing the long stream of events into a list of streams, each containing events generated while a single symbol was presented. The parsed data was then saved to a file in a different binary format using the Python pickle library (pickle protocol 5).

The acquisition order was randomized, and shuffling was reapplied for each acquisition session. The test samples (10,000) were randomly embedded within the sequence of training samples (60,000) during acquisition to mitigate potential biases developing during a single dataset acquisition period.

### Acquisition of EB vernier data

The acquisition of several EB datasets also incorporated Vernier stimuli. In these cases, the Vernier stimulus was presented on the computer display once every 100 repetitions. The camera motion and configuration during the Vernier repetition were identical to those used to acquire the source dataset symbols (MNIST, FMNIST, or KMNIST). This guaranteed that the Vernier data was acquired under the same conditions as the other tiny-symbol datasets. The vertical dimension of the Vernier edge was set to 28 display pixels to ensure spatial comparability with the other symbols displayed and sampled by the EB sensor. For each Vernier repetition, the horizontal offset in display pixels was chosen from the range of  ± 1 to  ± 7, where the sign determined the offset direction. The complete Vernier dataset included at least 50 repetitions for each of the offsets used.

### Estimating the display-to-photosensor-array projection ratio in pixels

We computed the size of a single display pixel projected onto the sensor (in sensor pixels) for the two distances used to acquire the datasets (D1 and D2). We used the camera’s magnification formula:

$$m=\frac{f}{s-f}$$

where *m* is the magnification, *f* is the lens’s focal length, and *s* is the distance between the computer display and the camera. To obtain the ratio in units of pix_display_/pix_sensor_, we multiplied *m* by the pitch_display_/pitch_sensor_ ratio, where *pitch* is the size of a single pixel. Given that we used a 4.5 mm focal-length lens, the DAVIS240C camera pixel size is 18.5 *μ*m, and the display pitch was 0.275 mm, the display pixel size to sensor pixel size ratio was 0.129 and 0.081 for D1 (s=525 mm) and D2 (s=830 mm), respectively. We also estimated the ratio for D1 empirically by measuring the distance in sensor pixels between two vertical lines displayed at a known horizontal distance on the display and projected onto the sensor with the camera positioned at the D1 distance. Using this method, we found that a single display pixel mapped to approximately $\sim \frac{1}{8}$ of a sensor pixel for the D1 configuration. This empirically estimated ratio of $\sim \frac{1}{8}$ is consistent with the calculation.

We note that, in practice, the optical and sensor responses are not perfectly confined to the nominally illuminated area: in retina-like event-based sensing, some residual spiking may occur outside the directly illuminated region, while in pixel-like frame-based sensing, intermediate gray levels may appear around the projected pixel (e.g., Fig. 1I). To account for these effects, as well as for the fact that a single display pixel is not an ideal point source on the screen, we adopted a slightly more conservative effective projection ratio in the subsequent analyses: $\frac{1}{7}$ instead of $\frac{1}{8}$.

### Model implementation and training details

**Transformer-based Model**: we employed a transformer-based architecture, similar to the Point Cloud Transformer^40^, for time-series classification. The input dimensionality was four (rather than three features per event or ’point’ in the Point Cloud Transformer) to account for two spatial dimensions (event’s x,y position on the sensor), one temporal dimension (event’s timestamp), and one polarity bit (event’s polarity). First, we shifted and scaled each feature element-wise by fixed offsets ( − 110,   − 35,   − 26,  0) and scalings (0.1,  1.0,  1.0,  1.0), resulting in approximately normalized data. We chose this fixed approximate normalization over per-batch or per-sample normalization to preserve relative spatio-temporal information. These processed inputs were then projected from dimension 4 to 256 via a learnable linear transformation.

The projected features were fed to *l* = 8 successive Transformer layers, each composed of a multi-head self-attention module with 4 heads (key/query dimension *d*_k_ = 256) and a position-wise MLP of dimension 512. We applied residual connections and layer normalization around both the attention and feedforward modules. A dropout rate of 0.1 was applied to the self-attention and feedforward sub-layers starting from layer 4.

For the final classification, we adopted an average-pooling head that computed the mean across events, followed by a fully connected projection of dimension 256. After another dropout step, a final linear layer produced logits for 10 output classes.

**GRU-based model**: we used a plain multilayer GRU with 14 layers to approximately match the parameter count of the PTr version. All other design choices were identical to the baseline PTr model: the input consisted of the event’s x,y position on the sensor, timestamp, and polarity. The classification head was identical to the one used with the PTr version and relied on the average activation of the last GRU layer over all time steps (i.e., average pooling). Input pre-processing, including centering and scaling, was also identical to the pre-processing used with the baseline model.

**Contrastive learning**: for contrastive pre-training^53^, we replaced the classification head with a projection head that projected into a 256-dimensional embedding space rather than a 10-dimensional classification space. It is common practice^53^ to use a projection head as a temporary set of neural network layers that transforms the features learned by the model into a space where similar examples are pulled together, and dissimilar ones are pushed apart-helping the model learn meaningful patterns without relying on labels. After this pre-training phase, we replaced the projection head with a classification head, which is the final set of layers designed to interpret these learned features and assign each input to a specific category. We then conducted supervised training, updating either the entire model or just the classification head, to optimize its performance as a classifier. We used Normalized Temperature-scaled Cross Entropy Loss with a temperature of 0.2 to train the model. In this method, cross-entropy loss is applied to logits computed as cosine similarities (scaled by temperature) between samples (see ref. ^53^ for details). We observed empirically that, for optimal performance, the ratio between positive and negative pairs should be small relative to ratios reported in the literature^53^. The positive/negative ratio was set to 5/64 on average, achieved using batches of 64 with 4/5 of the negative samples randomly dropped (this dropping was more time-efficient than working with smaller batches).

**Frame-based classifier**: the frame-based classifier reported in the main text is a ResNet-18 (PyTorch implementation). The training protocol (optimizer and learning-rate schedule) was similar to that used for the event-based trainings, except that we did not apply warm-up (i.e., the learning rate was high from the first epoch). Frame-based images (10 × 10 pixel frames, corresponding to an effective image size of approximately 5 × 5 pixels at D1) were interpolated to the standard 224 × 224 input size used by the ResNet family and intensity-normalized to the ImageNet red channel (mean = 0.485, std = 0.229) using PyTorch transforms. We used anti-aliasing during interpolation, as it empirically produced better results. Experiments with convolutional architectures trained directly on the original tiny frame-based images (without interpolation) resulted in lower accuracies.

As an alternative frame-based classifier, we explored a Vision Transformer (ViT) architecture. We used the publicly available ViT for MNIST^77^, increasing the default model size to more closely match the ResNet-18 parameter count (approx. 11M parameters). The best-performing configuration achieved 0.876 ± 0.0076 and 0.871 ± 0.022 (mean ± s.d.), for MNIST at D1, which is significantly lower than the performance of ResNet-18.

Optimization schedule: we trained the model for 100 epochs using a batch size of 64. We used the Adam optimizer with a maximum learning rate of 1 × 10^−4^, a linear warmup of 5 epochs with an initial rate of 1 × 10^−6^, and an exponential decay of the learning rate with a factor of 0.9 starting from epoch 40.

### Adding temporal noise (Jitter) to event-based datasets

We added temporal noise to the acquired data to estimate its effect on the network’s performance. The added noise for a single training instance was sampled from a normal distribution with a mean of 0 and a standard deviation chosen within the range of 0.1 to 2.0 ms.

When applied to training data, jitter values were sampled once for each event and used throughout the training (i.e., jitter was not resampled for each training epoch as is usually done when applying data augmentation), since we wanted to simulate a higher noise level at the sensor.

### Experimental repetitions, data splits, and statistical tests

We used five random seeds (i.e., variations in network initialization parameters and train/validation splits) to generate five training instances in all experiments; the same five seeds were used for all conditions. Development was performed with a validation set constituting 10% of the training samples. Trained models for which test accuracy is reported were trained excluding the validation set (the extra 10% that might have been used for training in these cases) to ensure consistency with the development results. The classification accuracy numbers mentioned in the paper correspond to the Mean ± Standard Deviation (STD) of the test set accuracy over these five different trained model instances for each experiment. The p-values for the comparisons between EB and FB models (Fig. 2F, last column), were calculated with the test set accuracies for the five EB instances and the corresponding five FB instances using a two-tailed Wilcoxon rank-sum test. The same test was used for comparing the no-jitter and jittered instances with 0.25 ms standard deviation.

### Reporting summary

Further information on research design is available in the Nature Portfolio Reporting Summary linked to this article.

## Supplementary information

Supplementary Information

Peer Review file

Reporting Summary

## Source data

Source Data 1

Source Data 2

## Data Availability

The datasets generated and used in this study (including tiny event-based and tiny frame-based versions) have been deposited in Zenodo and are publicly available at https://doi.org/10.5281/zenodo.20973756^39^. The three original FB datasets that were converted are also publicly available (MNIST^36^, Fashion-MNIST^37^, and Kuzushiji-MNIST^38^), as well as N-MNIST^30^. Source data are provided with this paper.

## Code availability

The code generated in this study is publicly available at Github (https://github.com/ahissar-lab/event-based-hyperacuity) and Zenodo (https://doi.org/10.5281/zenodo.20777652)^78^. The repository include also the in-house python code used for datasets collections and the python code used to generate the figures. The code can be used freely for academic research.
