# Supplementary material for: Temporal coding enables hyperacuity in event-based vision
Source: Nat Commun. 2026 Aug 20;17:9951. doi: 10.1038/s41467-026-76878-6 (PMC13586331; doi:10.1038/s41467-026-76878-6)
Supplement: Supplementary file 1 [file 41467_2026_76878_MOESM1_ESM.pdf]

Supplementary Material For the Article: Temporal coding enables hyperacuity in event based vision

Eldad Assa*, Alexander Rivkind*†, Michael Kreiserman, Fahad Shahbaz Khan, Salman Khan & Ehud Ahissar‡

August 19, 2026

*Equal contribution
†Corresponding author
‡Corresponding author

Metric	Human Eye	DVS Sensor	Comments
Sensor/receptor size [arcmin]	0.5 [1]	15	For camera we used: $\frac{\text{60 deg field of view}}{\text{240 sensors}} = 15$'
Minimal acquisition time [ms]	25 - 100 [2]; 40 [3]	20 - 80	Humans: data from masking experiments and primate electrophysiology. DVS sensor: Fig. 1G in the main text.
Typical velocity [arcmin/sec]	20-80 [4]	1800	Humans: range across subjects and conditions. DVS data: Fig. 1C
Typical velocity [receptors/sec]	40-160	120	computed based on the above figures
Typical number of crossed receptors during minimal acquisition time [receptors]	1-16	2.4 - 9.6	computed based on the above figures
Firing rate [Hz]	20-100	84	For humans, see Fig. 3B in [5]. For DVS sensor, according to event statistics (Fig. 1), the median inter-spike interval is $0.33msec$. Corresponding frequency is $1/0.33ms = 3KHz$. This should be divided by approximate image size of 6×6. Leading to a firing rate of 84.17 Hz per photoreceptor.

Table S1: **Comparison of key metrics between the human visual system and the DVS experimental setting.** Human minimal acquisition time is estimated from masking-based recognition studies and non-human primate electrophysiology, which together approximate the visual processing window sufficient to generate a meaningful downstream signal for recognition or top-down feedback. Firing-rate estimates are based on retinal physiology for stimuli of comparable size and shape. Combined with known intersaccadic eye-movement speeds, these data indicate that the DVS setting provides a reasonable approximation both the image smear caused by sensor motion and the spike count generated within a recognition-relevant temporal window. DVS values are reported for a sequence of 48 events acquired at camera distance D1.

Dataset	Distance	N events	N Runs	Accuracy Mean	Accuracy STD	Accuracy SEM
MNIST	D1	8.000	5	0.418	0.003	0.001
FMNIST	D1	8.000	5	0.471	0.005	0.003
MNIST	D2	8.000	5	0.416	0.002	0.001
KMNIST	D1	8.000	5	0.393	0.001	0.000
KMNIST	D2	8.000	5	0.362	0.003	0.001
FMNIST	D2	8.000	5	0.473	0.002	0.001
KMNIST	D1	16.000	5	0.605	0.002	0.001
FMNIST	D2	16.000	5	0.659	0.002	0.001
MNIST	D2	16.000	5	0.651	0.002	0.001
KMNIST	D2	16.000	5	0.559	0.003	0.001
FMNIST	D1	16.000	5	0.659	0.001	0.000
MNIST	D1	16.000	5	0.715	0.002	0.001
MNIST	D1	32.000	5	0.884	0.003	0.002
FMNIST	D1	32.000	5	0.767	0.002	0.001
MNIST	D2	32.000	5	0.795	0.003	0.002
KMNIST	D1	32.000	5	0.804	0.002	0.001
KMNIST	D2	32.000	5	0.672	0.001	0.001
FMNIST	D2	32.000	5	0.732	0.002	0.001
FMNIST	D2	48.000	5	0.759	0.001	0.001
KMNIST	D2	48.000	5	0.711	0.001	0.001
KMNIST	D1	48.000	5	0.847	0.003	0.001
MNIST	D2	48.000	5	0.840	0.002	0.001
FMNIST	D1	48.000	5	0.801	0.002	0.001
MNIST	D1	48.000	5	0.907	0.001	0.001
KMNIST	D2	64.000	5	0.740	0.006	0.003
FMNIST	D2	64.000	5	0.770	0.001	0.001
KMNIST	D1	64.000	5	0.864	0.001	0.001
MNIST	D2	64.000	5	0.853	0.002	0.001
MNIST	D1	64.000	5	0.924	0.002	0.001
FMNIST	D1	64.000	5	0.814	0.002	0.001
MNIST	D1	96.000	5	0.935	0.002	0.001
FMNIST	D1	96.000	5	0.829	0.004	0.002
KMNIST	D2	96.000	5	0.773	0.003	0.001
KMNIST	D1	96.000	5	0.879	0.001	0.001
MNIST	D2	96.000	5	0.847	0.005	0.002
FMNIST	D2	96.000	5	0.777	0.001	0.000
MNIST	D1	128.000	5	0.939	0.002	0.001
MNIST	D2	128.000	5	0.810	0.005	0.003
KMNIST	D1	128.000	5	0.886	0.004	0.002
FMNIST	D1	128.000	5	0.834	0.001	0.001
FMNIST	D2	128.000	5	0.777	0.003	0.002
KMNIST	D2	128.000	5	0.786	0.003	0.001

Table S2: Accuracy over event count: tabular results.

mode	N events	Jitter [ms]	N Runs	Mean	STD	SEM
jitter both	48	0.000	5	0.907	0.002	0.001
jitter both	48	0.100	5	0.904	0.002	0.001
jitter both	48	0.250	5	0.895	0.001	0.001
jitter both	48	0.500	5	0.881	0.003	0.001
jitter both	48	1.000	5	0.851	0.003	0.001
jitter both	48	2.000	5	0.805	0.002	0.001
jitter both	96	0.000	5	0.935	0.002	0.001
jitter both	96	0.100	5	0.933	0.001	0.000
jitter both	96	0.250	5	0.929	0.002	0.001
jitter both	96	0.500	5	0.918	0.002	0.001
jitter both	96	1.000	5	0.900	0.003	0.001
jitter both	96	2.000	5	0.862	0.003	0.001
jitter training	48	0.000	5	0.907	0.002	0.001
jitter training	48	0.100	5	0.906	0.002	0.001
jitter training	48	0.250	5	0.902	0.001	0.001
jitter training	48	0.500	5	0.895	0.001	0.000
jitter training	48	1.000	5	0.875	0.001	0.000
jitter training	48	2.000	5	0.840	0.002	0.001
jitter training	96	0.000	5	0.935	0.002	0.001
jitter training	96	0.100	5	0.935	0.001	0.000
jitter training	96	0.250	5	0.933	0.001	0.000
jitter training	96	0.500	5	0.928	0.002	0.001
jitter training	96	1.000	5	0.918	0.001	0.001
jitter training	96	2.000	5	0.892	0.001	0.000
jitter test	48	0.000	5	0.907	0.002	0.001
jitter test	48	0.100	5	0.903	0.003	0.001
jitter test	48	0.250	5	0.884	0.002	0.001
jitter test	48	0.500	5	0.819	0.006	0.003
jitter test	48	1.000	5	0.675	0.008	0.004
jitter test	48	2.000	5	0.487	0.012	0.005
jitter test	96	0.000	5	0.935	0.002	0.001
jitter test	96	0.100	5	0.934	0.001	0.001
jitter test	96	0.250	5	0.921	0.002	0.001
jitter test	96	0.500	5	0.877	0.006	0.003
jitter test	96	1.000	5	0.757	0.010	0.004
jitter test	96	2.000	5	0.551	0.011	0.005

Table S3: Adding temporal jitter: tabular results

Dataset	Distance	Mode	N Runs	Accuracy Mean	Accuracy STD	Accuracy SEM	Accuracy Change
MNIST	D1	contrastive finetune head	5	0.897	0.001	0.000	-0.010
MNIST	D1	contrastive XL finetune head	5	0.907	0.002	0.001	0.000
MNIST	D1	reference	5	0.907	0.001	0.001	0.000
MNIST	D1	contrastive finetune all	5	0.921	0.002	0.001	0.014
MNIST	D1	contrastive XL finetune all	5	0.929	0.001	0.001	0.022
MNIST	D2	contrastive finetune head	5	0.769	0.002	0.001	-0.071
MNIST	D2	contrastive XL finetune head	5	0.792	0.003	0.001	-0.048
MNIST	D2	reference	5	0.840	0.002	0.001	0.000
MNIST	D2	contrastive finetune all	5	0.845	0.002	0.001	0.005
MNIST	D2	contrastive XL finetune all	5	0.851	0.002	0.001	0.011
FMNIST	D1	contrastive finetune head	5	0.777	0.001	0.000	-0.024
FMNIST	D1	contrastive XL finetune head	5	0.787	0.002	0.001	-0.014
FMNIST	D1	reference	5	0.801	0.002	0.001	0.000
FMNIST	D1	contrastive finetune all	5	0.806	0.003	0.002	0.005
FMNIST	D1	contrastive XL finetune all	5	0.815	0.002	0.001	0.014
FMNIST	D2	contrastive finetune head	5	0.717	0.003	0.001	-0.042
FMNIST	D2	contrastive XL finetune head	5	0.731	0.002	0.001	-0.028
FMNIST	D2	reference	5	0.759	0.001	0.001	0.000
FMNIST	D2	contrastive finetune all	5	0.762	0.003	0.002	0.003
FMNIST	D2	contrastive XL finetune all	5	0.769	0.002	0.001	0.010
KMNIST	D1	contrastive finetune head	5	0.781	0.005	0.002	-0.066
KMNIST	D1	contrastive XL finetune head	5	0.807	0.001	0.001	-0.040
KMNIST	D1	reference	5	0.847	0.003	0.001	0.000
KMNIST	D1	contrastive finetune all	5	0.864	0.002	0.001	0.017
KMNIST	D1	contrastive XL finetune all	5	0.878	0.003	0.001	0.031
KMNIST	D2	contrastive finetune head	5	0.633	0.002	0.001	-0.078
KMNIST	D2	contrastive XL finetune head	5	0.650	0.003	0.001	-0.061
KMNIST	D2	reference	5	0.711	0.001	0.001	0.000
KMNIST	D2	contrastive finetune all	5	0.723	0.003	0.001	0.012
KMNIST	D2	contrastive XL finetune all	5	0.737	0.002	0.001	0.026

Table S4: Contrastive learning: tabular results

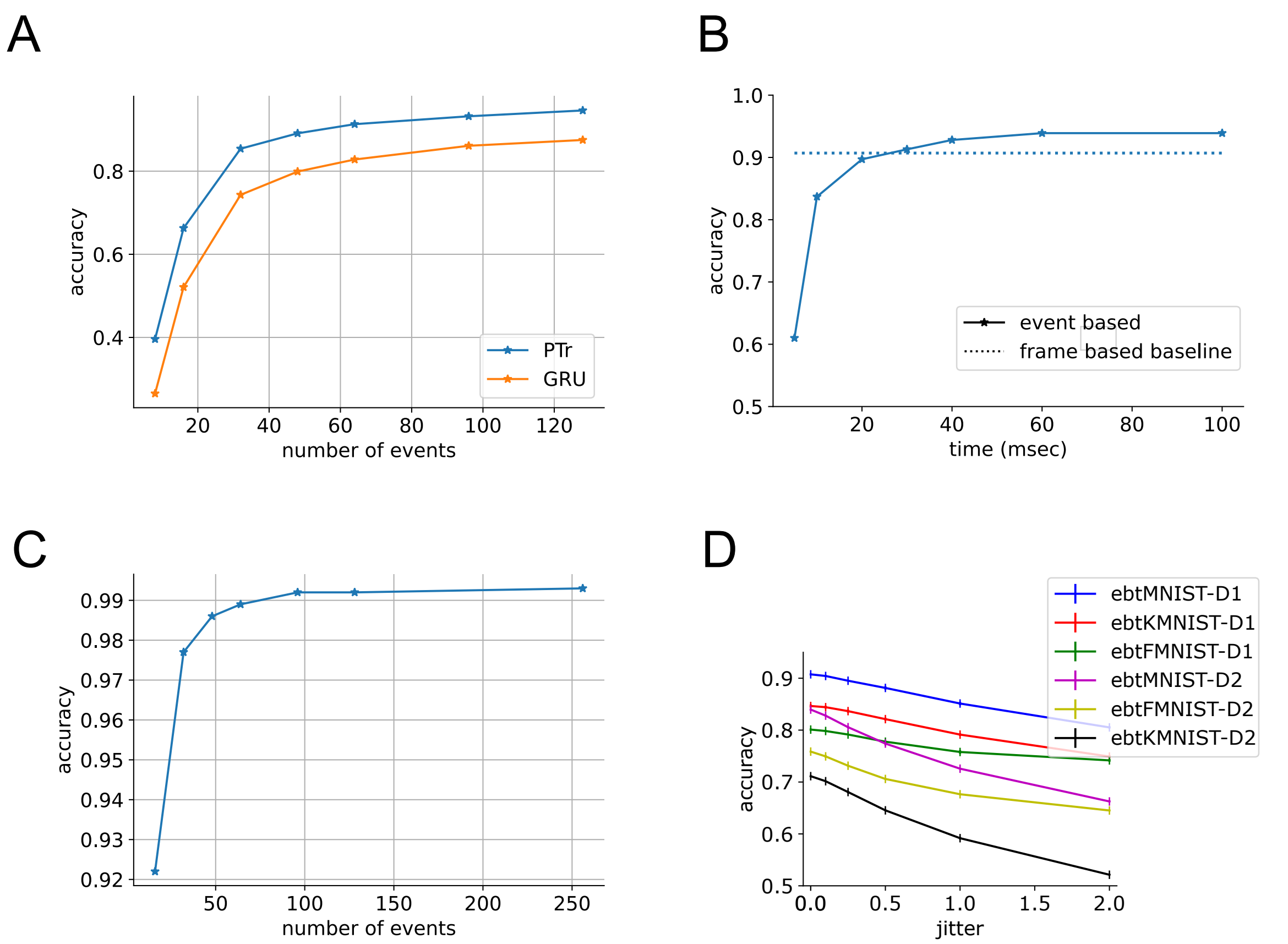

Figure S1: **Additional evaluations. A. Point Transformer vs. Recurrent Neural Network** GRU (Methods for details) and Point transformer (PTr) with comparable number of parameters and training times are shown (parameter count: ~ 5.9M for PTr, ~ 5.6M for GRU; evaluated at a development version of the ebtMNIST-D1). **B. Event based with controlled time window** Same as Fig. 2A, but with controlled parameter being events acquisition duration (i.e. all events occurred within a fixed time-window were use as input to the model) rather than event count. **C. N-MNIST** accuracy over different event counts. **D. Effect of temporal jitter** is shown here for all our datasets. The jitter was applied to both test and training sets. Source data are provided as a Source Data file.

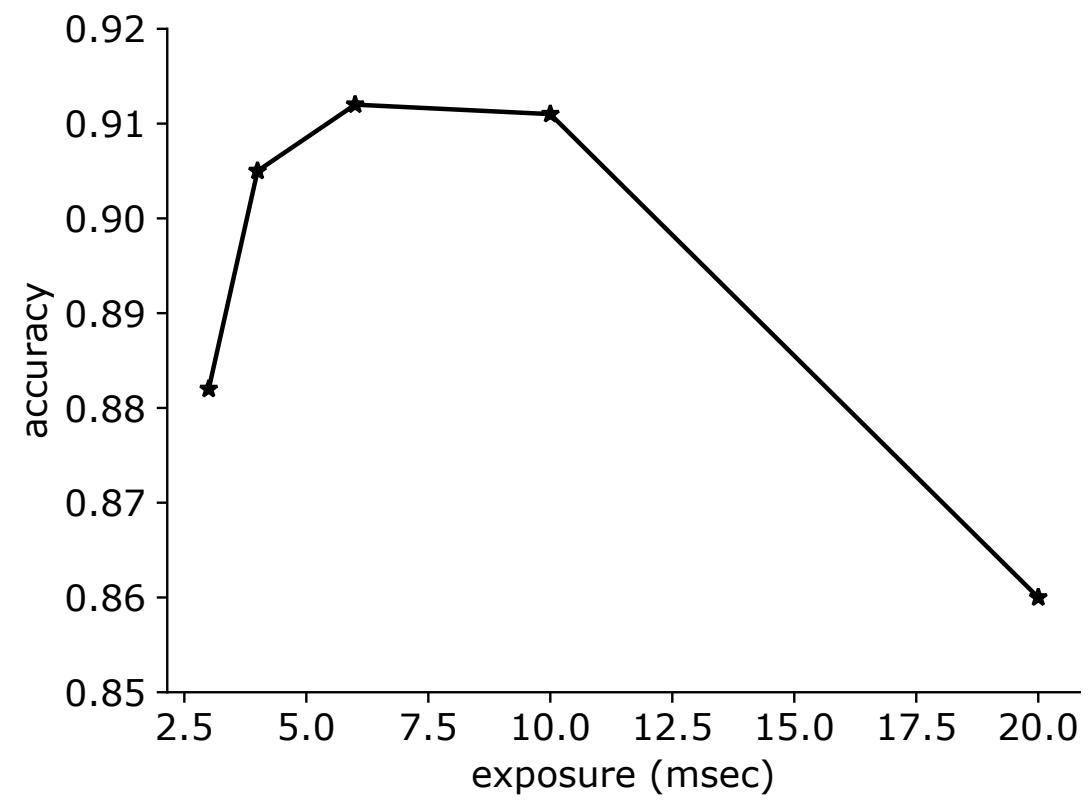

Figure S2: **FB model performance as a function of frame exposure time.** ResNet18 classification accuracy for models trained with FB datasets (fbtMNIST-D1) acquired with different exposure times. Based on these results we chose to used exposure time of 6 msecs for acquisition of all FB datasets. The accuracy value for 6 msec exposure is a bit different than the one used in the main text for fbtMNIST-D1 since setup was re-assembled and re-calibrated after the datasets used here were acquired, and before all EB and FB datasets used in the main text were acquired. Source data are provided as a Source Data file.

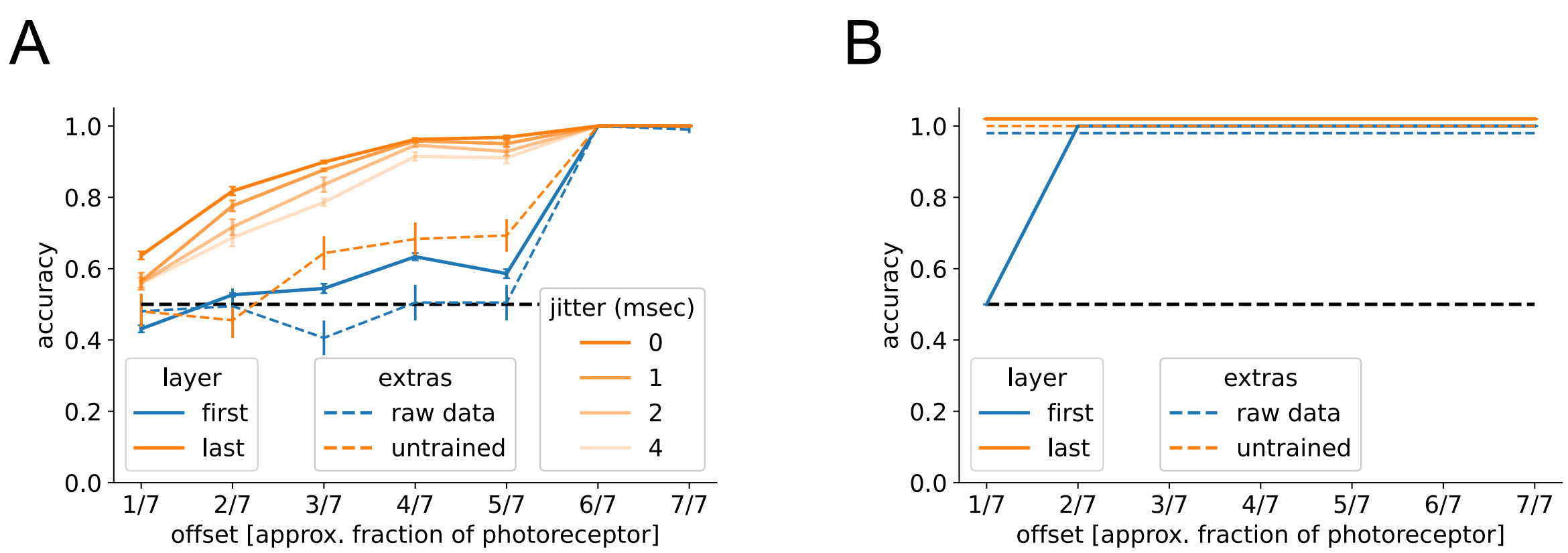

Figure S3: **Comparing Vernier learning in event-based and frame-based models.** We explored whether Vernier hyperacuity similarly emerges in the top layers of trained frame-based (FB) models. **A.** Fig. 4D, repeated with the addition of (i) results from the last layer of a fresh, untrained model and (ii) results for the raw stimulus. **B.** The same analysis for an FB model. (Jitter is not applicable to the FB case and is therefore omitted. The overlapping curves at accuracy = 1 are slightly offset for clarity. To accommodate the additional data, the figure is shown as a line plot rather than a bar plot.) It can be clearly seen that the effect is already present in the raw data as well as in the untrained network. This suggests that, in the FB system, hyperacuity cannot be attributed to learning. See the main text for our reservations about the feasibility of an stationary frame-based system. Source data are provided as a Source Data file.

References

[1] J. Hirsch and C. A. Curcio, "The spatial resolution capacity of human foveal retina," *Vision research*, vol. 29, no. 9, pp. 1095–1101, 1989.

[2] H. Tang, M. Schrimpf, W. Lotter, C. Moerman, A. Paredes, J. Ortega Caro, W. Hardesty, D. Cox, and G. Kreiman, "Recurrent computations for visual pattern completion," *Proceedings of the National Academy of Sciences*, vol. 115, no. 35, pp. 8835–8840, 2018.

[3] V. A. Lamme and P. R. Roelfsema, "The distinct modes of vision offered by feedforward and recurrent processing," *Trends in neurosciences*, vol. 23, no. 11, pp. 571–579, 2000.

[4] M. Rucci and J. D. Victor, "The unsteady eye: an information-processing stage, not a bug," *Trends in neurosciences*, vol. 38, no. 4, pp. 195–206, 2015.

[5] X. Pitkow, H. Sompolinsky, and M. Meister, "A neural computation for visual acuity in the presence of eye movements," *PLoS biology*, vol. 5, no. 12, p. e331, 2007.
