# Supplementary material for: Temporal coding enables hyperacuity in event-based vision
Source: Nat Commun. 2026 Aug 20;17:9951. doi: 10.1038/s41467-026-76878-6 (PMC13586331; doi:10.1038/s41467-026-76878-6)
Supplement: Supplementary file 2 [file 41467_2026_76878_MOESM2_ESM.pdf]

nature portfolio

Peer Review File

Temporal Coding Enables Hyperacuity in Event Based Vision

Corresponding Author: Professor Ehud Ahissar

This file contains all reviewer reports in order by version, followed by all author rebuttals in order by version.

Version 0:

Reviewer comments:

Reviewer #1

(Remarks to the Author)
In this report, the authors argue that temporal information in event-based (EB) image data (as opposed to frame-based image data; FB) enables accurate recognition of barely recognizable low-resolution images. Specifically, the modeling reported here shows that heavily pixelated images can be classified effectively using a 4-D temporal data space (with a decline in performance when said information is reduced). The authors further demonstrate that when trained in this way (EB over FB), netowkr performance shows signs of human-like Vernier hyperacuity. Regarding impact, the authors argue that the results of their modeling offer new perspectives on visual perception and offer new directions for "…advancing neuroscience, neuromorphic engineering, and energy efficient AI vision systems."

Overall, I found the topic of this manuscript to be interesting and very timely with respect to advancements in AI detection and recognition based on image-level input – specifically within the realm of remote sensing. However, I don't see how this work would either appeal to, let alone inform, the neuroscience community outside of sensory processing (and even that is tenuous). Further, in its current form, the manuscript will be quite impenetrable to a general audience as much of the methodology requires a significant background in the field of computer vision, and the various motivations behind many of the follow-up analyses are not explained. That's not to say that the work presented here isn't good – the results certainly argue for promising alternative strategies in remote sensing, it's just that I don't see much impact beyond that.

1) In the introduction, it's clear that aim of this study was inspired by the temporal sampling strategy of the early human visual system – specifically, those related to visual sensory processes in the subcortical visual pathways to seem to have evolved to boost the resolution limits of the retinae. However, it is important to note that gathering information is not synonymous with perception or recognition. While sampling information with as high resolution as possible is an important first step, the majority of perceptual processes rely on previous experience (or priors if you subscribe to the Bayesian perspectives on perception) to 'resolve' instances of a lack of clarity. Further, if we're considering very small, hardly recognizable objects, then we need to consider the task of the perceptual system is to map something that is poorly sampled to a higher resolution representation. That is, the perceptual system – as far as we know – does not learn low resolution representations, but instead, maps what is available to what has already been learned from higher resolution experiences. For example, I need to know what an E is before I can make sense of a poorly sampled version (even if slightly improved by drift). Further, seeing as how recognition and classification are the focus of this study, it is unlikely that vernier acuity plays any serious role there – the role of that form of acuity is likely to have more to do with differentiating spatial localization than stimulus classification. All that said, the entire manuscript should be revised to motivate the connections between neuromorphic learning strategies (the primary focus of this study) and biological vision as being largely sensory. There is nothing about perception that is incorporated into this study, so it seems that the authors have oversold the true impact and focus of this study.

2) Related to my previous comment training a network with poorly sampled images does not in any way map onto how the human brain learns about objects in the environment (excepting the first few months of development when the outer segments of the photoreceptors have yet to form a tightly packed array). High resolution objects (typically viewed up close) are most likely learned first, then those prior representations are used to make guesses about degraded information (until more information can be gathered). From what I can tell, the classifiers were trained on pixelated images, so all it is really learning is a representational space of novel shapes without (e.g., Figure 1I). While EB data certainly seem to help with that process, what the network is doing has no real biological relevance. That is, at no point (again, from what I can tell based on what is described – see my next comment about that) is there an attempt to map what is 'viewed' (and learned) to what the pixelated objects actually are, which is the true challenge of vision (the larger image tests are not the same thing here). What is presented here is simply improved discrimination in silico. Don't get me wrong, that is an interesting and important topic,

but one that mostly relevant to remote sensing, not biological vision.

3) The various training and analysis protocols that are presented in the Results section are poorly described (from a general audience standpoint). Aside from the EB/FB sampling – that part was clear and should be a model for how everything else is written. There are multiple variations of training protocols that are only vaguely laid out (the Method section only lays out parameter spaces...with little discussion of why those parameters were chosen I might add) without any guidance as to why they're being carried out (and the motivation for each of them). Further, many of the follow-up analyses were dropped on the reader without much (if any) explanation for what they were being run and what they do to contextualize the primary findings.

(Remarks on code availability)

Reviewer #2

(Remarks to the Author)
In this manuscript, the authors show that a spatio-temporal model incorporating precise temporal information outperforms frame-based models lacking temporal information in hyperacuity tasks on tiny still images captured by an event-based (EB) camera. The experimental procedures and results demonstrate the importance of temporal encoding for hyperacuity in computer vision. I believe that this study addresses important research questions, but additional analyses and justification are needed to improve the clarity and impact of the manuscript.

Major comments
1. Since this work aims to explain aspects of biological vision, it is necessary to show whether human participants exhibit similar behavior under the same experimental setup. In human experiments, the authors could use exposure time equivalent to the number of EB events and compare participants' microsaccades and classification accuracy to those of the EB camera and the PTr classifier.
2. The authors should justify the criterion used to set the frame-based (FB) exposure duration. The 4 ms exposure is considerably shorter than the time required for the EB camera to generate 48 events. The authors need to clarify whether the FB exposure duration is indeed comparable to that of the EB data.
3. To demonstrate that the observed Vernier hyperacuity arises from spatio-temporal information rather than spatial information alone, Vernier results obtained with the FB data should be included.
4. Although accuracy decreases when 2 ms of temporal jitter is added, some conditions still exceed the FB classification accuracy (e.g., Fig. 2C, the 96-event jittered-training condition). Given that 2 ms greatly exceeds the median inter-event interval, one would expect a more substantial accuracy drop if temporal precision were critical. The authors need to explain why performance remains robust under this level of jitter.
5. The authors need to report the statistical significance of differences between conditions (for example, in Fig. 2A, between the FB baseline and the corresponding EB accuracy).

Minor comments
1. Page 2: In "source dataset x distance," use the multiplication sign "×" instead of the letter "x."
2. Page 5: Use "participants" rather than "subjects."
3. Page 16, Figure 1D–F: Add labels indicating the corresponding original symbol digits.
4. Page 18, Figure 3A: Add labels indicating that the upper and lower plots correspond to different stimuli.
5. Consider adding panel titles to guide readers (e.g., in Fig. 2A and 2B, add "D1," "D2" at the top of the panels).

(Remarks on code availability)

Reviewer #3

(Remarks to the Author)
The authors investigate the role of precise temporal coding in object recognition, using event-based cameras that move across static, tiny images. The motivation stems from an analogy to biological vision, which they argue similarly relies on fine temporal dynamics. Their central claim is that precise temporal information—available in event-based systems—can significantly enhance recognition accuracy, particularly for small images where spatial information is limited. This study contributes to a growing body of work that emphasizes the importance of temporal dynamics in artificial vision systems, though several conceptual and methodological clarifications are necessary.

Major Comments

1. Framing and Novelty of Contribution
1.1. The introduction dedicates substantial space to connecting event-based vision with biological vision. While this is a useful framing, much of the introduction covers well-established ideas, supported by references (lines 54–57) that seem to come late in the introduction. In my opinion, the authors should consider streamlining this section to focus more quickly on the specific research question and their novel contribution.
1.2. The key question posed is how precise temporal coding facilitates image recognition. The authors argue that their primary contribution is showing this in the context of tiny images, where temporal dynamics provide critical cues. However,

the principle they present—temporal coding enhancing recognition—should apply equally to larger images. What makes tiny images a uniquely informative case for this hypothesis? This needs further justification.

2. Comparison to Frame-Based Temporal Methods
A crucial omission is the lack of comparison with temporally enriched frame-based approaches. Temporal information can also be introduced in conventional cameras by moving the frame or camera, thereby generating a sequence of frames. If the claim is about the superiority of event-based vision, a direct comparison to a frame-based temporal method is warranted. If instead the goal is to demonstrate that temporal information is beneficial (regardless of the sensor type), this should be made more explicit and carefully distinguished from prior work (e.g., Rivera-Rubio et al., ICIAP 2013; Lagorce et al., 2016).

3. Model Architecture Confounds
3.1 The Frame-Based approach uses ResNet-18, which was originally designed for higher-resolution images. Since the dataset comprises tiny images, did the authors modify the architecture (e.g., filter sizes) accordingly?
3.2 Moreover, the Event-Based model uses a transformer-based architecture, which is more elaborate than ResNet-18. To ensure a fair comparison, a transformer or equally complex model should also be tested on the Frame-Based input.

4. Biological Relevance Claims
4.1 The authors suggest (line 78) that their results reinforce the importance of temporal coding in biological vision. However, the paper does not present any biological studies directly demonstrating this point. Such a statement needs to be supported or rephrased to reflect the evidence presented.
4.2 In line 84, the authors mention simulating the retina's response. This is vague. Biological retina responses—especially in primates—are high-dimensional, with 25–30 different feature-selective channels. The authors should clarify what specific aspects of retinal computation are being simulated or approximated.

Minor Comments
Typo in Reference 24

(Remarks on code availability)

Reviewer #4

(Remarks to the Author)
In this paper, Eldad Assa and colleagues used event-based (EB) sensing (retina-like, mimicking miniature eye motion) to investigate the question if spatial information and visual acuity (i.e., visual resolution) can be improved beyond the sensor sampling density, particularly when comparing spatiotemporal to purely spatial (frame-based, FB) coding. To evaluate the data (derived from common image datasets) a transformer-based model is applied with variations in supervised, self-supervised, and unsupervised training. The authors show convincingly that their system outperforms purely FB strategies due to the processing of precise temporal input.
Overall, even though the general idea is not new and the results are somewhat expected, the study demonstrates a valuable straightforward implementation of current state-of-the-art hardware and software, which could trigger further important research and technical applications.

I have 2 major concerns that should be addressed.

1)
The study is inspired by biological vision as the authors set out in their introduction, claiming that the underlying mechanisms of spatiotemporal encoding remain unclear. I wonder why the authors do not consider well-known spatiotemporal asymmetries in on/off pathways (starting in the retina). The Alonso group has done substantial work in this direction (e.g., Kremkow et al., PNAS, 2014) and another group demonstrated temporal asymmetries in cortical activation upon abrupt changes in stimulus luminance polarity, providing a temporal code for sensitive contrast detection of object borders (Rekauzke et al., 2014).

2)
Given that the bio-inspired approach of this study and the attempt to improve our understanding of natural vision (while enhancing the development of technical systems that are ultimately capable of autonomous interpretation, e.g. classification, of natural images), the used data set does not convincingly prove that the results would generalize towards more naturalistic input scenarios. The authors conclude to have used "relatively simple datasets" and emphasize a need "to extend this research to more complex and diverse real-world visual tasks". To my opinion it would be important for a general readership of Nature Communications to provide such data and go beyond a "proof of principle".

Minor comments:

A “standard hyperacuity experiment” was performed (line 243) using “Vernier-like stimuli” (line 221). To my understanding in standard hyperacuity experiments vertical lines with spatial offsets are most commonly used. The authors should explain why they used another variant.

The authors might cite (Rucci et al., Nature, 2007) as this original work showed how miniature eye movements enhance fine spatial detail.

Unclear why ref. #75 is cited (line 241) in the context of visual processing.

Legend/Figure 2 “F Information from panels A,B presented in tabular form“. – Unclear how data points (information?) in A,B translate to the tab.

Figure C and D. – Line types (jitter_test and jitter_training) are hard to differentiate.

Legend/Fig. 3B “train” – Should be training? trained?

Legend/Fig. 4D– typo “chace” level

“It has been hypothesized that the visual system may counteract smearing through spatial interpolation [1, 12] or statistical inference [13], or bypass it by processing temporal delays between retinal spikes [9, 14, 15, 16] …“ (line 32)
Another possible mechanism to counteract motion smear would be backward suppression (Chemla et al. 2019) below perceptual thresholds - as motion smear activity may still contain useful information (Geisler, 1999) at low amplitudes (Jancke, 2000).

(Remarks on code availability)

Version 1:

Reviewer comments:

Reviewer #1

(Remarks to the Author)
The authors have successfully addressed my concerns.

(Remarks on code availability)

Reviewer #2

(Remarks to the Author)
The authors have adequately addressed my major comments 2 through 5, and their rationale for why human experiments cannot be conducted is reasonable. The comparisons between biological and artificial setups are also sufficiently presented.
However, my concern regarding the scope of the biological claims remains. The evidence, while technically sound, is insufficient to fully account for biological vision, which limits the manuscript's appeal to the broader neuroscience community. The authors might consider reframing the contribution more explicitly toward the computer vision community, rather than positioning the work as an explanation of biological vision. Such a reframing would sharpen the manuscript's focus and better align its claims with the evidence presented.
That said, this shift in framing would necessarily narrow the target audience. Given the current scope and framing of the manuscript, I believe it would find a better fit in a more specialized journal.

(Remarks on code availability)

Reviewer #3

(Remarks to the Author)
My main comment is that the manuscript is framed heavily around understanding mechanisms of biological hyperacuity, as it comes across from the introduction. The presented experiments are entirely computational, relying on event-camera data and transformer-based models. The work incorporates some biologically inspired design choices—such as simulated fixational eye movements, approximate matching of temporal and spatial scales. However, these elements primarily serve as parameter choices or arguments rather than direct empirical validation, and no direct link is established to neural

processing or perception.

The main result—that precise temporal information is sufficient to decode hyperacuity-level spatial differences in an engineered vision system—is interesting and clearly demonstrated, and is consistent with known properties of biological vision such as the benefits of temporal integration and motion-induced sampling. However, it remains unclear what this implies for biological vision, where encoding mechanisms, noise characteristics, and downstream decoding constraints differ substantially. In particular, the emergence of hyperacuity-like behavior in a learning-based model operating on event-based data should not be taken as evidence that similar decoding strategies are used in the retina or early visual system.

As such, the work is best interpreted as a proof-of-principle demonstration of computational sufficiency under temporal encoding rather than as evidence for mechanisms underlying biological hyperacuity. Clarifying this distinction, or strengthening the connection to biological data or mechanistic models, would improve the manuscript. The paper is likely to be of strong interest to the computer vision and event-based sensing communities, but its relevance to biological vision is more limited, and the framing could be adjusted accordingly.

(Remarks on code availability)

Reviewer #4

(Remarks to the Author)
The authors have largely addressed my concerns and I am happy that they also found the suggested literature helpful and relevant.

I have one final comment related to the authors' reply "We thank the Reviewer for this important comment. We have now added these two relevant references to the Discussion (Lines 358-363):"

Line 302-307: The current phrasing implies that the mentioned references are exclusively about "higher-level perceptual inference or cortical learning" rather than related to "sensory encoding", which I think is not the case (e.g., learning is not addressed at all in these references).

Reference 55 is about visual spatial resolution and deals with mechanisms that are inherited from the retina. Additionally, to my understanding, reference 56 is much closer to the idea presented in the current study than the authors suggest with their current statement.

Specifically, Rekauzke et al. showed how a simultaneous counterchange in luminance is translated into asynchronous activation, thus, transforming a spatial event (i.e, luminance change at a stimulus border) into a temporal event. Their study suggests that temporal asymmetries may "influence perception of shape whenever abrupt shifts in visual objects or gaze cause counterchange of luminance at high-contrast borders".

This resonates very much with the idea of EB visual sensors that "… generate events asynchronously, when individual photosensors detect luminance changes [21], unlike frame-based (FB) cameras that collect synchronous information at fixed rates." (line 51-53).

Altogether, I think both references above should be more highlighted in the context of the ideas of the current study and at least ref. 56 should be mentioned also in the introduction.

(Remarks on code availability)

Open Access This Peer Review File is licensed under a Creative Commons Attribution 4.0 International License, which permits use, sharing, adaptation, distribution and reproduction in any medium or format, as long as you give appropriate credit to the original author(s) and the source, provide a link to the Creative Commons license, and indicate if changes were made.
In cases where reviewers are anonymous, credit should be given to 'Anonymous Referee' and the source.
The images or other third party material in this Peer Review File are included in the article's Creative Commons license, unless indicated otherwise in a credit line to the material. If material is not included in the article's Creative Commons license and your intended use is not permitted by statutory regulation or exceeds the permitted use, you will need to obtain permission directly from the copyright holder.
To view a copy of this license, visit https://creativecommons.org/licenses/by/4.0/

Point-by-Point answer to reviewers

The Line numbers specified below refer to the file eb_visual_hyperacuity-tracked_changes.pdf (uploaded)

REVIEWER COMMENTS

Reviewer #1 (Remarks to the Author):

In this report, the authors argue that temporal information in event-based (EB) image data (as opposed to frame-based image data; FB) enables accurate recognition of barely recognizable low-resolution images. Specifically, the modeling reported here shows that heavily pixelated images can be classified effectively using a 4-D temporal data space (with a decline in performance when said information is reduced). The authors further demonstrate that when trained in this way (EB over FB), network performance shows signs of human-like Vernier hyperacuity. Regarding impact, the authors argue that the results of their modeling offer new perspectives on visual perception and offer new directions for "…advancing neuroscience, neuromorphic engineering, and energy efficient AI vision systems."

Overall, I found the topic of this manuscript to be interesting and very timely with respect to advancements in AI detection and recognition based on image-level input – specifically within the realm of remote sensing. However, I don't see how this work would either appeal to, let alone inform, the neuroscience community outside of sensory processing (and even that is tenuous). Further, in its current form, the manuscript will be quite impenetrable to a general audience as much of the methodology requires a significant background in the field of computer vision, and the various motivations behind many of the follow-up analyses are not explained. That's not to say that the work presented here isn't good – the results certainly argue for promising alternative strategies in remote sensing, it's just that I don't see much impact beyond that.

Reply:
We thank the Reviewer for their constructive review. We agree that the direct scope of our study is sensory encoding rather than higher-level perception, and we have revised the manuscript to make this distinction explicit (see also our response to the next comment). At the same time, we note that hyperacuity is a cross-modal principle, observed not only in vision but also in tactile and auditory systems, and that sensory encoding directly affects downstream processing. To reflect this, we have modified the opening paragraph of the Introduction to refer to hyperacuity in other modalities and to clarify why understanding encoding mechanisms at the sensory organ is foundational for downstream processing (Lines 23-29). In addition, we note that visual motion-induced hyperacuity was reported in humans also in recognition tasks (recognition of small opto-types; Intoy et. al, 2020, Ref #47 in the manuscript).

We also took seriously the reviewer's concern about accessibility. We have revised the Methods and Results sections to provide clearer motivation for each analysis, simplified technical phrasing where possible, and improved figure labeling. These changes should make the manuscript more readable for a broad neuroscience audience while keeping the technical rigor needed for computer vision researchers. Please see our Reply to the Reviewer's Comment #3 below for a list of modifications made in order to address these issues.

To make the paper more accessible to the general audience we also followed the Reviewer's suggestion and added explanations of the motivations to run specific follow-up analyses and experiments. In lines 140-144 we explain the motivation to run the analysis described in Figure 2A,B,F; the motivation for the analysis depicted in Figure 2C,D is explained in lines 191-195. The justification for the three different conditions used in Figure 2C,D was added in lines 207-211. In lines 242-245 we briefly explain the motivation to run the analysis described in Figure 3 and In lines 294-298 we explain the motivation to run the experiment described in Figure 4.

1) In the introduction, it's clear that aim of this study was inspired by the temporal sampling strategy of the early human visual system – specifically, those related to visual sensory processes in the subcortical visual pathways to seem to have evolved to boost the resolution limits of the retinae. However, it is important to note that gathering information is not synonymous with perception or recognition. While sampling information with as high resolution as possible is an important first step, the majority of perceptual processes rely on previous experience (or priors if you subscribe to the Bayesian perspectives on perception) to 'resolve' instances of a lack of clarity. Further, if we're considering very small, hardly recognizable objects, then we need to consider the task of the perceptual system is to map something that is poorly sampled to a higher resolution representation. That is, the perceptual system – as far as we know – does not learn low resolution representations, but instead, maps what is available to what has already been learned from higher resolution experiences. For example, I need to know what an E is before I can make sense of a poorly sampled version (even if slightly improved by drift). Further, seeing as how recognition and classification are the focus of this study, it is unlikely that vernier acuity plays any serious role there – the role of that form of acuity is likely to have more to do with differentiating spatial localization than stimulus classification. All that said, the entire manuscript should be revised to motivate the connections between neuromorphic learning strategies (the primary focus of this study) and biological vision as being largely sensory. There is nothing about perception that is incorporated into this study, so it seems that the authors have oversold the true impact and focus of this study.

Reply:
We have revised the entire manuscript to address the points raised by the Reviewer, as detailed below and in our replies to the other comments. We agree that the scope of our work is limited to sensory encoding and not to higher-level perceptual inference or cortical learning. To make this clear, we added the following statement to the Discussion (Lines 358-363): *"At the same time, it is important to clarify the scope of our conclusions. Our study addresses sensory encoding only; we do not model higher-level perceptual inference or cortical learning. Our*

experiments were designed to explore a specific component of sensory mechanism: the contribution of temporal precision in input signals that are undersampled spatially. We acknowledge that the biological visual system likely employs a variety of mechanisms, including spatial, spatiotemporal and polarity-based ones [86,87] to achieve accurate perception."

To better motivate the use of tiny images and clarify their role in revealing the contribution of temporal encoding, we added the following paragraph (Lines 58-63): *"Tiny images constitute a particularly stringent test case in which spatial information alone is insufficient: the object covers only a few sensor pixels, so fine spatial details that are required in order to recognize the object are eliminated by pixelation on the sensor. In this regime, even small temporal delays across neighboring pixels, induced by drift or motion, create discriminative cues that a static representation cannot capture [8]. Thus, tiny images expose the contribution of precise temporal encoding in a way that larger images, which already contain abundant spatial information, do not."*

Regarding Vernier offsets, we appreciate the Reviewer's point that this task relates more to spatial localization than to classification. In our manuscript, we use the Vernier paradigm not as a model of recognition but as a sensitive probe of sub-pixel encoding, showing that hyperacuity-like readouts emerge naturally once temporal information is exploited. We have clarified this distinction in the text (Results, Lines 294-298; Discussion, Lines 343-344).

2) Related to my previous comment training a network with poorly sampled images does not in any way map onto how the human brain learns about objects in the environment (excepting the first few months of development when the outer segments of the photoreceptors have yet to form a tightly packed array). High resolution objects (typically viewed up close) are most likely learned first, then those prior representations are used to make guesses about degraded information (until more information can be gathered). From what I can tell, the classifiers were trained on pixelated images, so all it is really learning is a representational space of novel shapes without (e.g., Figure 1I). While EB data certainly seem to help with that process, what the network is doing has no real biological relevance. That is, at no point (again, from what I can tell based on what is described – see my next comment about that) is there an attempt to map what is 'viewed' (and learned) to what the pixelated objects actually are, which is the true challenge of vision (the larger image tests are not the same thing here). What is presented here is simply improved discrimination in silico. Don't get me wrong, that is an interesting and important topic, but one that is mostly relevant to remote sensing, not biological vision.

Reply:

We thank the Reviewer for another important comment, We now explicitly state the Reviewer's observation in the manuscript, indicating the potential relevance of our in silico observations to biological vision (Discussion, Lines 405-416): "*Another central direction for future work is the development of a more biologically plausible curriculum of learning: humans and animals may learn to perceive high-resolution views before generalizing to degraded inputs, whereas our networks were trained directly on undersampled images. This design emphasizes the role of temporal coding under conditions of minimal spatial information. Importantly, however, the*

present work shows that ANNs using EB data as an input can learn useful representations in an unsupervised manner (Fig. 3C,D), based solely on the temporal constancy of the input. This suggests that, in the more general case where recognition is not restricted to tiny images, generalization from typical images to very small ones may be supported by such learned representations. In other words, even if the learner is not routinely exposed to meaningful tiny images, the feature space required to process them may still emerge through unsupervised learning and be recruited when needed. Future work combining high- and low-resolution training could more closely capture biological learning dynamics."

In general, we would like to stress that we disagree that our results are not relevant to biological vision, as detailed in our responses above. In particular, we contend that demonstrating the importance of temporal coding for recognising tiny objects (FIig. 2) provides strong support for a viable candidate model of biological vision.

3) The various training and analysis protocols that are presented in the Results section are poorly described (from a general audience standpoint). Aside from the EB/FB sampling – that part was clear and should be a model for how everything else is written. There are multiple variations of training protocols that are only vaguely laid out (the Method section only lays out parameter spaces...with little discussion of why those parameters were chosen I might add) without any guidance as to why they're being carried out (and the motivation for each of them). Further, many of the follow-up analyses were dropped on the reader without much (if any) explanation for what they were being run and what they do to contextualize the primary findings.

Reply:
We have modified the Results and Methods sections and added details and motivations as required by the Reviewer. We expect that these changes will indeed make the paper more accessible to non-experts:

1. Section 2.2 in the original manuscript was split to two sections. New Section 2.2- *Information Collected by Event-based and Frame-based Sensing of Tiny Images;* and new Section 2.3- *Accurate Event Timing Contains Task-Relevant Information*. We added a paragraph at the beginning of Section 2.2 (Lines 140-144) to better explain our general approach and our motivation in using ANNs: "*In order to assess the amount of information acquired in each sensing mode, we trained ANN models to classify the recorded samples. The performance of these trained models served as a benchmark for the information contained in the data. Thus, the models were not selected for their biological relevance but for their ability to decode information effectively.".* In addition, we extended the following paragraphs (Lines 145-169) and added text to explain and justify the specific models that were selected for each sensing mode.
2. In several places along the manuscript we have added explanations of the motivations to run specific follow-up analyses and experiments (see also our first Reply above). In lines 140-144 we explain the motivation to run the analysis described in Figure 2A,B,F;

the motivation for the analysis depicted in Figure 2C,D is explained in lines 191-195. The justification for the three different conditions used in Figure 2C,D was added in lines 207-211. In lines 242-245 we briefly explain the motivation to run the analysis described in Figure 3 and In lines 294-298 we explain the motivation to run the experiment described in Figure 4.

3. Parameters selection and tuning: we have added the following paragraph to Section 2.2 (Lines 160-166): "*Model's hyperparameters (e.g. learning rate, number of layers and layers' size) were determined through manual fine-tuning, supplemented by a partial grid search, using event-based MNIST with 48 events at distance D1. The only parameter adjusted for other datasets was the spatial centering (x, y coordinates). For the frame-based models, we used PyTorch's built-in implementation of ResNet-18, with the frame-based input rescaled accordingly. We also experimented with manually designed convolutional neural network architectures tailored to our frame-based data, but these yielded poorer performance (2–3\% lower test accuracy; results not shown).*"

We also made the project's code and the data we collected publicly available, including the code defining the models and the training procedures. This was done to enable anyone interested to replicate the analyses described in the manuscript and to evaluate the effects of modifying specific parameters.

Reviewer #2 (Remarks to the Author):

In this manuscript, the authors show that a spatio-temporal model incorporating precise temporal information outperforms frame-based models lacking temporal information in hyperacuity tasks on tiny still images captured by an event-based (EB) camera. The experimental procedures and results demonstrate the importance of temporal encoding for hyperacuity in computer vision. I believe that this study addresses important research questions, but additional analyses and justification are needed to improve the clarity and impact of the manuscript.

Major comments
1. Since this work aims to explain aspects of biological vision, it is necessary to show whether human participants exhibit similar behavior under the same experimental setup. In human experiments, the authors could use exposure time equivalent to the number of EB events and compare participants' microsaccades and classification accuracy to those of the EB camera and the PTr classifier.

Reply:
We agree with the Reviewer that our findings, which are based on experiments conducted with an artificial setup, should ultimately be validated by testing them in human experiments. Two major considerations affect the design of such experiments: 1. They require real time closed-loop gaze contingent stimulus delivery at an extremely high accuracy in order to be able

to stimulate a very small number of receptors. In our recent attempt we could control the stimulus such that it generated, according to our best assessment, more than 2000 retinal spikes during the period required for the subject to perform a recognition task at 60% success, where chance level was 20% {1} (see Ref. {2} for spike count assessment). 2. Such tasks are very hard to perform by human subjects {1}. Furthermore, despite choosing experimental parameters which resulted in biologically relevant numbers in a few key variables, as presented in Table S1, many more degrees of freedom should be added to our artificial system to better simulate the biological visual system in order for such a comparison to be meaningful and conclusive (added to manuscript, see below).

Given these constraints, we cannot envision a human experiment that can address the concern of the Reviewer with currently available technology. However, we suggest that the human experiments we have already done provide valuable information in this respect. Gruber & Ahissar {1} concluded that the visual system actively controls specific oculomotor variables to match stimulus conditions - in that experiment the human visual system was shown to maintain the drift speed constant across changes in image size. Their data further suggested that the controlled speed maintained minimal inter-receptor delays in the fovea around 1 ms, which matches temporal resolutions of neural processing {3,4}. This result is consistent with the human visual system counting on retinal temporal coding of image shapes.

Additional evidence from previous studies is available for two key parameters that affect recognition (i.e. classification) in our reduced artificial setup: 1. The spike count needed for recognition; this can be evaluated from estimated firing rates {5} together with estimated time {6,7} required for recognition, and 2. the spatial spread induced by eye movement {8}. We have now added this supporting evidence to the manuscript (Table S1, see below).

Given these constraints and previous literature, we have addressed the Reviewer's comment as follows. First, we have added in the Discussion an explicit mention of the difference in degrees of freedom between the biological and our artificial systems (Lines 434-438). Second, we now compare the biological and artificial constraints that are relevant to our study, in more detail. We point this out in the main text (Line 136) and elaborate in Table S1 and its caption:
" Human minimal acquisition time is estimated from masking-based recognition studies and non-human primate electrophysiology, which together approximate the visual processing window sufficient to generate a meaningful downstream signal for recognition or top-down feedback. Firing-rate estimates are based on retinal physiology for stimuli of comparable size and shape. Combined with known intersaccadic eye-movement speeds, these data indicate that the DVS setting provides a reasonable approximation of both the image smear caused by sensor motion and the spike count generated within a recognition-relevant temporal window."

References:

{1} Gruber, L.Z. and E. Ahissar, Closed loop motor-sensory dynamics in human vision. PLOS ONE, 2020. 15(10): p. e0240660.

{2} Gruber, L.Z., S. Ullman, and E. Ahissar, Oculo-retinal dynamics can explain the perception of minimal recognizable configurations. Proceedings of the National Academy of Sciences, 2021. 118(34): p. e2022792118.

{3} Meister, M. and M.J. Berry, 2nd, The neural code of the retina. Neuron, 1999. 22(3): p. 435-50.

{4} Jones, L.M., et al., Robust temporal coding in the trigeminal system. Science, 2004. 304(5679): p. 1986-9.

{5} Pitkow, X., H. Sompolinsky, and M. Meister, A neural computation for visual acuity in the presence of eye movements. PLoS Biol, 2007. 5(12): p. e331.

{6} Tang, H., et al., Recurrent computations for visual pattern completion. Proceedings of the National Academy of Sciences, 2018. 115(35): p. 8835-8840.

{7} Lamme, V.A. and P.R. Roelfsema, The distinct modes of vision offered by feedforward and recurrent processing. Trends in neurosciences, 2000. 23(11): p. 571-579.

{8} Rucci, M. and J.D. Victor, The unsteady eye: an information-processing stage, not a bug. Trends in Neurosciences, 2015. 38: p. 195-206.

2. The authors should justify the criterion used to set the frame-based (FB) exposure duration. The 4 ms exposure is considerably shorter than the time required for the EB camera to generate 48 events. The authors need to clarify whether the FB exposure duration is indeed comparable to that of the EB data.

Reply:

We thank the reviewer for raising this important aspect. To address this comment, we systematically explored the effect of exposure time on frame-based classification accuracy and a performed re-acquisition of datasets in optimized conditions, as detailed below.

We evaluated the performance of frame-based datasets with frame exposure time that are comparable to the typical event-based acquisition time (20 msecs for ebtMNIST-D1 and 30 msecs for ebtMNIST-D2). These exposure times resulted in reduced performance: 0.860±0.002 and 0.652±0.003 compared to 0.882±0.001 and 0.664±0.002 for the original exposure time used (for D1 and D2 respectively; mean±std).

Furthermore, we systematically explored the effect of exposure time on frame-based classification accuracy for fbtMNIST-D1. The top plot below depicts the accuracies of FB models trained on fbtMNIST-D1 acquired with different exposure times (the plot was also added to manuscript supplementary material- Figure S2). Based on these results the optimal value, which was found to be 6 msecs, was used to re-acquire all other frame-based datasets.

The datasets used eventually to train the networks (both FB and EB) and evaluate their accuracies were re-acquired after reassembling and calibrating the setup again. EB datasets were re-acquired also with the new conditions to allow fair comparison between FB and EB conditions, as control during calibration over other parameters were improved. The FB

MNIST-D1 accuracy which appears in the main text is slightly different from the one in the plotted black line (0.907±0.001 vs. 0.912±0.001) as a result of the different configuration used, this difference doesn't affect any of our conclusions.

The bottom plot and the table (revised Figure 2A,I) display the accuracies of the EB models as a function of the number of events used for training and inference for the different datasets, and the FB models accuracies using the same configuration (dashed lines in the bottom plot). Significantly better accuracies for the EB models compared to the FB models are indicated by large circles in the plot and a bold font in the table.

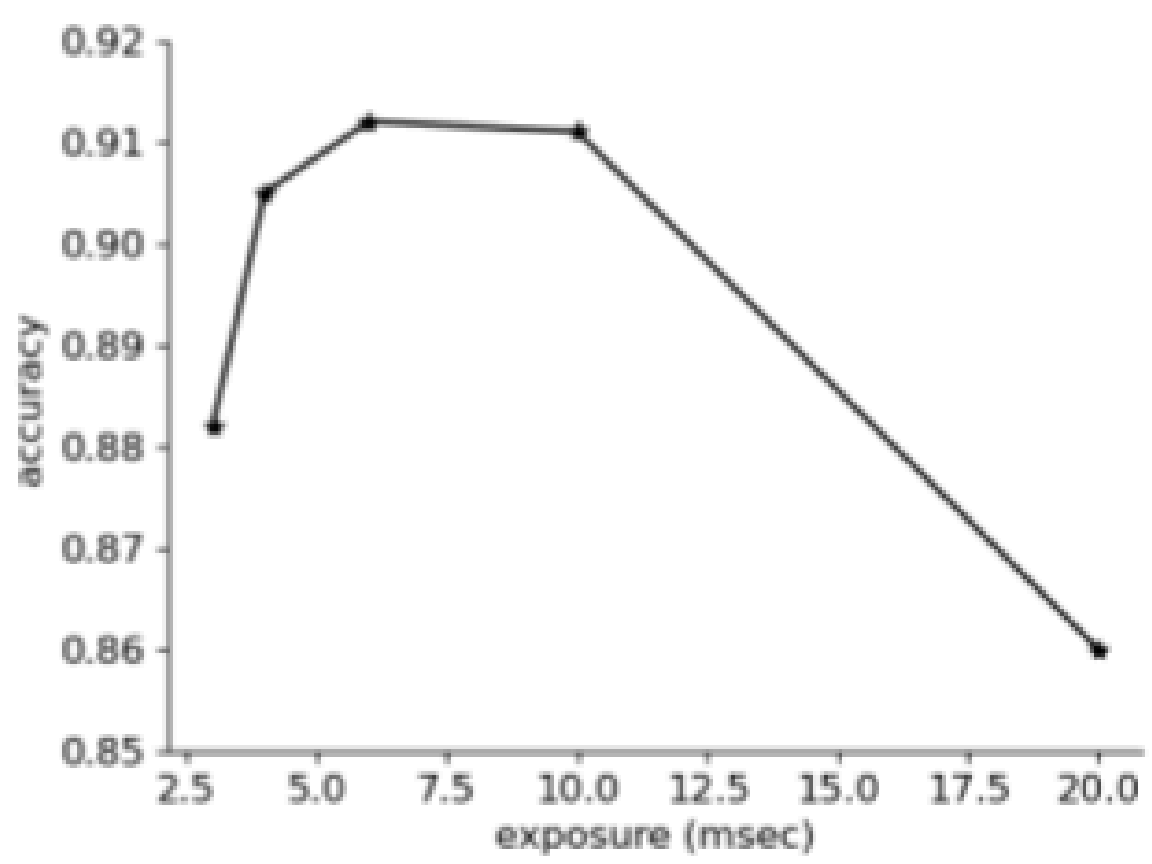

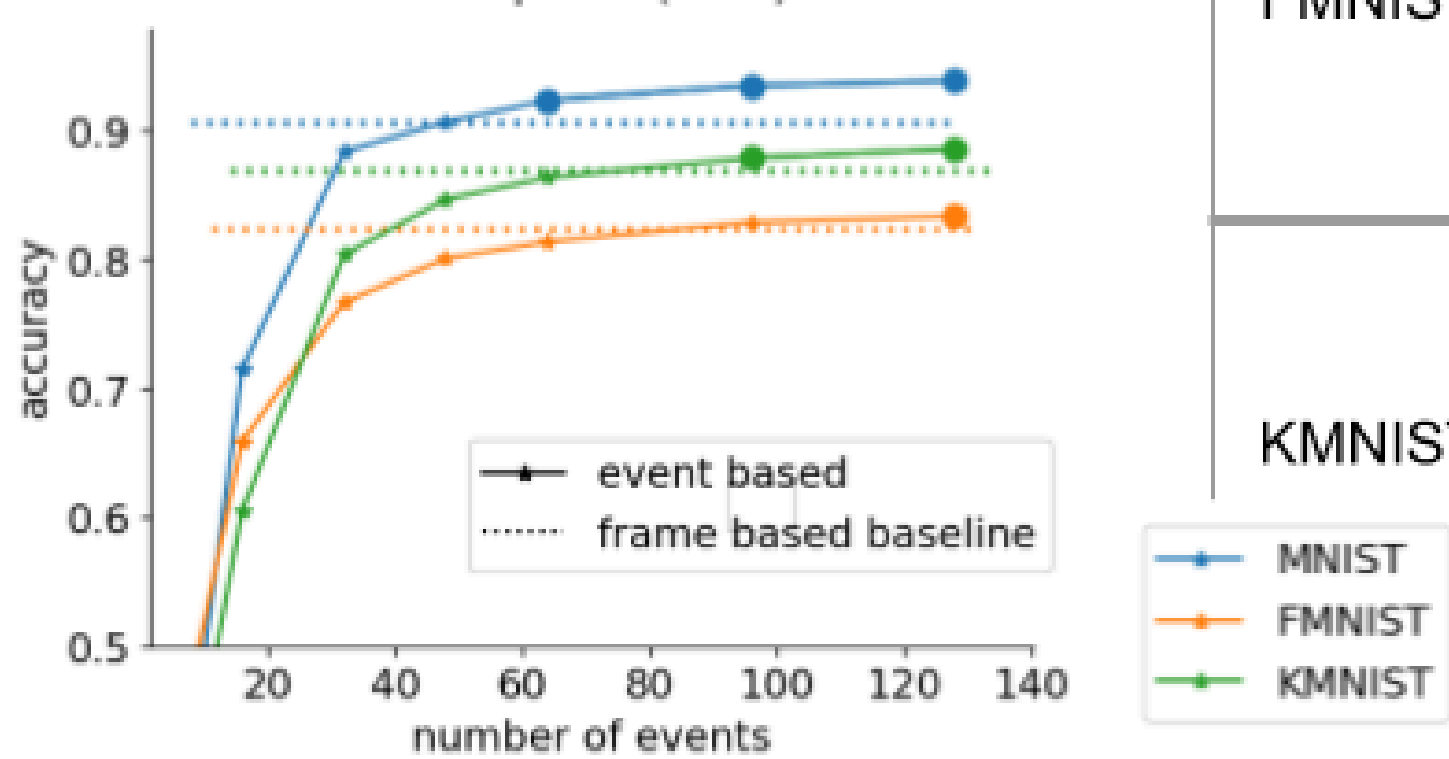

DS	input	D1	D2
MNIST	eb (48)	0.907	**0.840**
	eb (96)	**0.935**	**0.847**
	fb (6 ms)	0.907	0.703
FMNIST	eb (48)	0.801	**0.759**
	eb (96)	0.829	**0.777**
	fb (6 ms)	0.824	0.739
KMNIST	eb (48)	0.847	**0.711**
	eb (96)	**0.879**	**0.773**
	fb (6 ms)	0.870	0.706

3. To demonstrate that the observed Vernier hyperacuity arises from spatio-temporal information rather than spatial information alone, Vernier results obtained with the FB data should be included.

Reply:
We thank the reviewer for raising this important point. In the revised manuscript, we addressed it in **two complementary ways**.

First, as requested, we added the **FB Vernier analysis** (new Supplementary Fig. S3). However, we would like to clarify that, in our setup, the FB condition is not the most selective test of whether Vernier performance depends on temporal coding. The newly added FB analysis shows

that performance is already high for the raw FB stimulus, and remains high even for an untrained network, indicating that this condition does not isolate any learned mechanism.

Second, and more importantly, **following the reviewer's concern about the temporal aspect** (revised Fig 4D), we added a new temporal-jitter analysis for the EB Vernier task, which was not included in the original submission. We believe this is the fairer and more direct test of temporal coding. The reason is that jitter selectively perturbs the timing of events while preserving the general mechanism of decoding from events rather than from a static bitmap. Thus, unlike the FB comparison, the jitter manipulation isolates the contribution of temporal precision itself. In the revised manuscript, we show that adding temporal jitter significantly degrades Vernier performance in the EB condition. This shows that the classifier is not relying on spatial information alone, but on the precise spatio-temporal structure of the event stream.

We therefore included the FB results for completeness, in direct response to the reviewer's request (Supplementary Figure S3), but we respectfully suggest that the newly added EB jitter experiment (Figure 4D) is the more decisive control for the reviewer's underlying question: whether the observed Vernier hyperacuity arises from temporal coding rather than from residual spatial cues alone.

4. Although accuracy decreases when 2 ms of temporal jitter is added, some conditions still exceed the FB classification accuracy (e.g., Fig. 2C, the 96-event jittered-training condition). Given that 2 ms greatly exceeds the median inter-event interval, one would expect a more substantial accuracy drop if temporal precision were critical. The authors need to explain why performance remains robust under this level of jitter.

Reply:

We thank the Reviewer for highlighting this important point, which bears directly on our central claim regarding the contribution of temporal information.

Our interpretation is that the relatively strong performance under the 2 ms jitter condition reflects redundancy in longer event sequences, rather than a lack of dependence on precise timing. In general, increasing the number of events provides more opportunities for the classifier to detect informative temporal structure, and therefore increases robustness to noise. Under a simple linear-decoding framework with additive noise, doubling the sequence length while holding noise variance constant would increase the signal-to-noise ratio by a factor of two. Equivalently, a substantially larger noise level would be required to produce the same degradation in performance. Although our decoder is non-linear, the same qualitative intuition should still apply: longer sequences are expected to be more tolerant to temporal perturbation (and this is indeed the case as follows from Fig. 2C that the Reviewer mentioned) .To conclude, under jitter manipulation, precise temporal relationships are degraded probabilistically, not abolished.

In application to our claim, if decoding depends on local constellations of spikes with sub-millisecond precision (as Fig. 2C suggests), jitter will reduce the probability that any given constellation is preserved, but this effect is partially offset when more events are available. In longer sequences, enough informative temporal patterns can remain intact for classification to stay above FB accuracy, even though performance is reduced relative to the non-jittered condition.

We would also like to mention that with the newly acquired EB and FB datasets it is not the case anymore that the 96-events jittered-training exceeds the FB classification accuracy (0.892±0.001 vs. 0.907±0.001)

On the other hand, we find the fact that jitter with std as small as 0.25 ms, which is smaller than the median IEI (0.33 ms), resulted in a significant (although small) drop in performance (Lines 204-206) to be a strong indication for utilization of precise temporal information by the model.

5. The authors need to report the statistical significance of differences between conditions (for example, in Fig. 2A, between the FB baseline and the corresponding EB accuracy).

Reply:
Statistical tests (Wilcoxon rank-sum test) were applied for EB models vs. FB baselines. The cases where EB and FB performances were significantly different ($p < 0.05$) are now indicated in Figure 2A,B by large symbols (circles) and in Figure 2F by a bold font.

Minor comments
1. Page 2: In “source dataset x distance,” use the multiplication sign “×” instead of the letter “x.”
Reply: Done.
2. Page 5: Use “participants” rather than “subjects.”
Reply: Done.
3. Page 16, Figure 1D–F: Add labels indicating the corresponding original symbol digits.
Reply: The labels were added to Figure 1D–F.
4. Page 18, Figure 3A: Add labels indicating that the upper and lower plots correspond to different stimuli.
Reply: Done
5. Consider adding panel titles to guide readers (e.g., in Fig. 2A and 2B, add “D1,” “D2” at the top of the panels).
Reply: In Fig. 2A and 2B, “D1” and “D2” were added at the top of the panels. In addition titles were added also to Fig. 2C and 2E.

Reviewer #3 (Remarks to the Author):

The authors investigate the role of precise temporal coding in object recognition, using event-based cameras that move across static, tiny images. The motivation stems from an

analogy to biological vision, which they argue similarly relies on fine temporal dynamics. Their central claim is that precise temporal information—available in event-based systems—can significantly enhance recognition accuracy, particularly for small images where spatial information is limited. This study contributes to a growing body of work that emphasizes the importance of temporal dynamics in artificial vision systems, though several conceptual and methodological clarifications are necessary.

Major Comments

1. Framing and Novelty of Contribution

1.1. The introduction dedicates substantial space to connecting event-based vision with biological vision. While this is a useful framing, much of the introduction covers well-established ideas, supported by references (lines 54–57) that seem to come late in the introduction. In my opinion, the authors should consider streamlining this section to focus more quickly on the specific research question and their novel contribution.

Reply:

We thank the reviewer for the comment. The Introduction section was substantially modified. The two paragraphs discussing general EB vision background in original manuscript were merged to a single paragraph where almost all of the general background was removed including most of the references in lines 54-57 in the original submission. The introduction contains now a few paragraphs discussing the biological phenomena, the models suggested to explain the phenomena, a very short introduction to EB vision and how our approach is different from the approach taken by previous work in this field, and our research contribution.

1.2. The key question posed is how precise temporal coding facilitates image recognition. The authors argue that their primary contribution is showing this in the context of tiny images, where temporal dynamics provide critical cues. However, the principle they present—temporal coding enhancing recognition—should apply equally to larger images. What makes tiny images a uniquely informative case for this hypothesis? This needs further justification.

Reply:

We thank the Reviewer for raising this important point. We agree that, in the biological context, object recognition in general, including for larger objects, likely utilizes the temporal dynamics of sensory signals. Our focus on tiny images is motivated by the fact that they isolate and reveal the contribution of precise temporal encoding in a way that larger images, which already contain abundant spatial information, do not.

In the comparisons presented in the manuscript between artificial decoders (EB Transformer vs. FB CNN/Transformer), the advantage of event-based (EB) sensing is expected to emerge primarily in regimes where static spatial information alone is insufficient to fully solve the task, given that standard frame-based DNNs are already highly effective at extracting spatial features. This constraint makes tiny images a uniquely informative test case for our hypothesis.

We have added a paragraph to the manuscript to clarify this point (Lines 58-63): "*Tiny images constitute a particularly stringent test case in which spatial information alone is insufficient: the object covers only a few sensor pixels, so fine spatial details that are required in order to recognize the object are eliminated by pixelation on the sensor. In this regime, even small temporal delays across neighboring pixels, induced by drift or motion, create discriminative cues that a static representation cannot capture [12]. Thus, tiny images expose the contribution of precise temporal encoding in a way that larger images, which already contain abundant spatial information, do not.*"

2. Comparison to Frame-Based Temporal Methods
A crucial omission is the lack of comparison with temporally enriched frame-based approaches. Temporal information can also be introduced in conventional cameras by moving the frame or camera, thereby generating a sequence of frames. If the claim is about the superiority of event-based vision, a direct comparison to a frame-based temporal method is warranted. If instead the goal is to demonstrate that temporal information is beneficial (regardless of the sensor type), this should be made more explicit and carefully distinguished from prior work (e.g., Rivera-Rubio et al., ICIAP 2013; Lagorce et al., 2016).

Reply:
We thank the Reviewer for sharpening this point. We agree that there are ways to use conventional FB cameras such that the visual signal would include temporal information (e,g, processing multiple frames of the same scene with a moving camera; please see our ICLR 2022 paper for an example {1}). Specifically here we aim to illustrate the advantage of bio-inspired event-based acquisition compared to static single frame input which is the standard method for visual classification. We now make it clear in the Discussion (see below).

We would add that in most recent published cases where event-based visual input is being used to train a model, events are integrated over a time-window into a frame-like structure and precise temporal information is lost (e.g. {2}, {3}). An exception is the work of Lagorce et al.,{4} , which describes an unsupervised model for object recognition that learns spatio-temporal event-based features and enables propagation of precise time information through its layers. However, the datasets they used to benchmark their model didn't allow them to demonstrate its potential advantage as:

1. They couldn't demonstrate a performance-wise advantage of their model compared to standard frame-based models as they used small datasets where performance is saturated.
2. In the datasets they used, the spatial information seems sufficient to solve the classification task, as suggested by the time-collapsed EB frames presented in their paper; in these frames the stimulus is clearly visible, as was shown for similar datasets {5} and in contrast to our case (see our Fig. 1I),

In contrast, we describe an event-based model that reaches performance on-par with SOTA frame-based classifiers (and less than 100%) in the specific task of tiny symbol recognition

(Figure 2A,B,I), and isolate the specific contribution of precise temporal information by showing the models' accuracy sensitivity to added temporal jitter (Figure 2C,D)

To address the Reviewer's comment we have modified the section in the Discussion that compares our work with previous relevant EB studies, and added specific discussions of the point raised by the Reviewer: "*More generally, prior work has demonstrated that spatio-temporal information can contribute to recognition performance in both frame-based and event-based systems [95, 62, 86]*" (Lines 367-369) and "*Here we focused on a regime in which spatial information alone is insufficient. By considering tiny images, we isolate conditions under which temporal coding becomes critical for successful recognition, rather than merely advantageous. Accordingly, our results challenge the general assumption described above and differ from those showing that fine temporal resolution mainly contributes to precise motion compensation [56], for example, by "canceling" motion and generating static representations of a scene [17]. In our case (Figures 2,3,4), precise temporal information is used to construct representations rather than to compensate for perturbations, achieving superior performance compared to equivalent spatial frame-based models..*" (Lines 384-391).

References:

{1} Rivkind, A., Ram, O., Assa, E., Kreiserman, M., & Ahissar, E. (2021, September 29). *Visual hyperacuity with moving sensor and recurrent neural computations*. International Conference on Learning Representations. https://openreview.net/forum?id=p0rCmDEN_-

{2} Yousefzadeh, A., Orchard, G., Serrano-Gotarredona, T., & Linares-Barranco, B. (2018). Active Perception With Dynamic Vision Sensors. Minimum Saccades With Optimum Recognition. IEEE Transactions on Biomedical Circuits and Systems, 12(4), 927–939. IEEE Transactions on Biomedical Circuits and Systems. https://doi.org/10.1109/TBCAS.2018.2834428

{3} Torbunov, D., Ren, Y., Ghose, A., Dim, O., & Cui, Y. (2024). EvRT-DETR: The Surprising Effectiveness of DETR-based Detection for Event Cameras (No. arXiv:2412.02890). arXiv. https://doi.org/10.48550/arXiv.2412.02890

{4} Lagorce, X., Orchard, G., Galluppi, F., Shi, B. E., & Benosman, R. B. (2017). HOTS: A Hierarchy of Event-Based Time-Surfaces for Pattern Recognition. *IEEE Transactions on Pattern Analysis and Machine Intelligence*, *39*(7), 1346–1359. IEEE Transactions on Pattern Analysis and Machine Intelligence. https://doi.org/10.1109/TPAMI.2016.2574707

{5} Iyer, L. R., Chua, Y., & Li, H. (2021). Is Neuromorphic MNIST Neuromorphic? Analyzing the Discriminative Power of Neuromorphic Datasets in the Time Domain. Frontiers in Neuroscience, 15. https://www.frontiersin.org/articles/10.3389/fnins.2021.608567

3. Model Architecture Confounds

3.1 The Frame-Based approach uses ResNet-18, which was originally designed for higher-resolution images. Since the dataset comprises tiny images, did the authors modify the architecture (e.g., filter sizes) accordingly?

3.2 Moreover, the Event-Based model uses a transformer-based architecture, which is more elaborate than ResNet-18. To ensure a fair comparison, a transformer or equally complex model should also be tested on the Frame-Based input.

Reply:

We thank the Reviewer for this important comment. We added a Methods section with information about Visual transformer results and about interpolation to ResNet-18 dimensions (Lines: 581-594):

> "*The frame-based classifier reported in the main text is a ResNet-18 (PyTorch implementation). The training protocol (optimizer and learning-rate schedule) was similar to that used for the event-based trainings, except that we did not apply warm-up (i.e., the learning rate was high from the first epoch). Frame-based images (10×10 pixel frames, corresponding to an effective image size of approximately 5×5 pixels at D1) were interpolated to the standard 224×224 input size used by the ResNet family and intensity-normalized to the ImageNet red channel (mean = 0.485, std = 0.229) using PyTorch transforms. We used anti-aliasing during interpolation, as it empirically produced better results. Experiments with convolutional architectures trained directly on the original tiny frame-based images (without interpolation) resulted in lower accuracies. As an alternative frame-based classifier, we explored a Vision Transformer (ViT) architecture. We used the publicly available ViT for MNIST [Onken2025mnistvit], increasing the default model size to more closely match the ResNet-18 parameter count (approx. 11M parameters). The best-performing configuration achieved 0.876±0.0076 and 0.871± 0.022 (mean±s.d.) for MNIST at D1, which is significantly lower than the performance of ResNet-18.*"

4. Biological Relevance Claims

4.1 The authors suggest (line 78) that their results reinforce the importance of temporal coding in biological vision. However, the paper does not present any biological studies directly demonstrating this point. Such a statement needs to be supported or rephrased to reflect the evidence presented.

Reply:

Upon re-reading line 78, we agree that the statement could be interpreted as suggesting that the importance of temporal coding in biological vision has already been established. We have therefore removed this ambiguous sentence from the revised manuscript.

4.2 In line 84, the authors mention simulating the retina's response. This is vague. Biological retina responses—especially in primates—are high-dimensional, with 25–30 different feature-selective channels. The authors should clarify what specific aspects of retinal computation are being simulated or approximated.

Reply:
We agree. Following the Reviewer's comment we rephrased the sentence to more accurately reflect our experiment (Lines 115-116): "*We used an EB camera (DAVIS240C, iniVation AG; [63]) to acquire visual events of static objects sampled through sensor motion.*"

Minor Comments
Typo in Reference 24
Reply: Fixed

Reviewer #4 (Remarks to the Author):

In this paper, Eldad Assa and colleagues used event-based (EB) sensing (retina-like, mimicking miniature eye motion) to investigate the question if spatial information and visual acuity (i.e., visual resolution) can be improved beyond the sensor sampling density, particularly when comparing spatiotemporal to purely spatial (frame-based, FB) coding. To evaluate the data (derived from common image datasets) a transformer-based model is applied with variations in supervised, self-supervised, and unsupervised training. The authors show convincingly that their system outperforms purely FB strategies due to the processing of precise temporal input. Overall, even though the general idea is not new and the results are somewhat expected, the study demonstrates a valuable straightforward implementation of current state-of-the-art hardware and software, which could trigger further important research and technical applications.

I have 2 major concerns that should be addressed.

1)
The study is inspired by biological vision as the authors set out in their introduction, claiming that the underlying mechanisms of spatiotemporal encoding remain unclear. I wonder why the authors do not consider well-known spatiotemporal asymmetries in on/off pathways (starting in the retina). The Alonso group has done substantial work in this direction (e.g., Kremkow et al., PNAS, 2014) and another group demonstrated temporal asymmetries in cortical activation upon abrupt changes in stimulus luminance polarity, providing a temporal code for sensitive contrast detection of object borders (Rekauzke et al., 2014).

Reply:
We thank the Reviewer for this important comment. We have now added these two relevant references to the Discussion (Lines 358-363): *"At the same time, it is important to clarify the scope of our conclusions. Our study addresses sensory encoding only; we do not model higher-level perceptual inference or cortical learning. Our experiments were designed to explore a specific component of sensory mechanism: the contribution of temporal precision in input signals that are undersampled spatially. We acknowledge that the biological visual system likely*

employs a variety of mechanisms, including spatial, spatiotemporal and polarity-based ones [86,87] to achieve accurate perception."

2)

Given that the bio-inspired approach of this study and the attempt to improve our understanding of natural vision (while enhancing the development of technical systems that are ultimately capable of autonomous interpretation, e.g. classification, of natural images), the used data set does not convincingly prove that the results would generalize towards more naturalistic input scenarios. The authors conclude to have used "relatively simple datasets" and emphasize a need "to extend this research to more complex and diverse real-world visual tasks". To my opinion it would be important for a general readership of Nature Communications to provide such data and go beyond a "proof of principle".

Reply:

We thank the Reviewer for another important comment. We agree that extending this research to more complex and diverse real-world visual tasks will greatly benefit the field. However, we believe that such an extension should follow, rather than precede, a solid proof-of-principle demonstration. Our goal in this first paper is to establish the validity and explanatory power of the approach in a manner that will be convincing to the readership of *Nature Communications*. Once the proof of principle is firmly established, broader extensions will naturally follow.

In order to direct attention to this justified point, we have added references to naturalistic scenarios in the manuscript. First, we emphasized the naturalistic aspect of the Vernier stimulus used (edge instead of line, Lines 306-308): "*To better reflect naturalistic scenarios, we used the single-edge version of the Vernier task [83] rather than the more commonly used version with isolated bars or lines*." Second, we explicitly highlighted naturalistic scenarios as a direction for future work (Lines 401-404): "*Future work aimed at understanding biological vision could extend these findings to more biomimetic models, including asynchronous networks (SNNs) and biologically inspired learning rules, implemented on neuromorphic hardware [100, 101, 102], while generalizing to more naturalistic input scenarios.*" These additions complement our statement in the Concluding Remarks: "*We demonstrated these advantages on relatively simple datasets, highlighting the need to extend this research to more complex and diverse real-world visual tasks*".

Minor comments:

A "standard hyperacuity experiment" was performed (line 243) using "Vernier-like stimuli" (line 221). To my understanding in standard hyperacuity experiments vertical lines with spatial offsets are most commonly used. The authors should explain why they used another variant.

Reply:

We agree that vertical lines are more commonly used in such experiments, however the basic phenomena was also demonstrated with an edge Vernier {1,2}, which, as we said, reflects

better naturalistic scenarios. In our case, as the EB camera we use is sensitive to contrast changes, an edge Vernier is probably sufficient to illustrate the phenomena of sub-pixel temporal dependent information as illustrated in Figure 4B,C. We now refer to this issue in the manuscript, as mentioned above (Lines 306-308).

{1} Carney, T., & Klein, S. A. (1997). Resolution Acuity is better than Vernier Acuity. Vision Research, 37(5), 525–539. https://doi.org/10.1016/S0042-6989(97)85878-6

{2} Klein, S. A., Casson, E., & Carney, T. (1990). Vernier acuity as line and dipole detection. Vision Research, 30(11), 1703–1719. https://doi.org/10.1016/0042-6989(90)90154-D

The authors might cite (Rucci et al., Nature, 2007) as this original work showed how miniature eye movements enhance fine spatial detail.

Reply: A reference to Rucci 2007 was added in Lines 34-35.

Unclear why ref. #75 is cited (line 241) in the context of visual processing.

Reply: Reference to Carr removed - thank you.

Legend/Figure 2 “F Information from panels A,B presented in tabular form“. – Unclear how data points (information?) in A,B translate to the tab.

Reply: We replaced “... presented in a tabular form” to “... presented in a table”.

Figure C and D. – Line types (jitter_test and jitter_training) are hard to differentiate.

Reply: The graphics of the figure was improved to allow easier differentiation between the plotted lines. Differentiating symbols were enlarged, and differences in line types for the 3 conditions were made more pronounced.

Legend/Fig. 3B “train” – Should be training? trained?

Reply: The distinction there is between accuracy for the *train* set and the *validation* set. This is now clarified in the figure caption.

Legend/Fig. 4D– typo “chace” level

Reply: Figure 4D was revised and legend doesn’t include this line any more. Instead a sentence was added to the figure’s caption to explain the meaning of the dashed line.

“It has been hypothesized that the visual system may counteract smearing through spatial interpolation [1, 12] or statistical inference [13], or bypass it by processing temporal delays

between retinal spikes [9, 14, 15, 16] …“ (line 32)
Another possible mechanism to counteract motion smear would be backward suppression (Chemla et al. 2019) below perceptual thresholds - as motion smear activity may still contain useful information (Geisler, 1999) at low amplitudes (Jancke, 2000).

Reply:
Thank you. We have added Chemla et al (2019) to the relevant section (Line 47). We did not include an explanation based on the other two references, as this would fall outside the scope of our discussion.

RESPONSE TO REVIEWERS' COMMENTS

Reviewer #1 (Remarks to the Author):

The authors have successfully addressed my concerns.

Reply:

We thank the reviewer for their positive view on our revised version

Reviewer #2 (Remarks to the Author):

The authors have adequately addressed my major comments 2 through 5, and their rationale for why human experiments cannot be conducted is reasonable. The comparisons between biological and artificial setups are also sufficiently presented. However, my concern regarding the scope of the biological claims remains. The evidence, while technically sound, is insufficient to fully account for biological vision, which limits the manuscript's appeal to the broader neuroscience community. The authors might consider reframing the contribution more explicitly toward the computer vision community, rather than positioning the work as an explanation of biological vision. Such a reframing would sharpen the manuscript's focus and better align its claims with the evidence presented.
That said, this shift in framing would necessarily narrow the target audience. Given the current scope and framing of the manuscript, I believe it would find a better fit in a more specialized journal.

Reply:

We appreciate the Reviewer’s concern of potentially overestimating the biological relevance of our study. We made a number of changes to the manuscript to mitigate these concerns from two perspectives:

1. We carefully reframed any claims that may sound speculative or overestimate with relation to biological vision.
    a. We softened the framing in the Abstract, replacing stronger language implying direct biological convergence with a more limited statement that the artificial system shows “hyperacuity-like behavior” and that active event-based sensing can recover spatial detail using precise timing.

b. We revised the Introduction to remove a broad claim that the work clarifies biological mechanisms of hyperacuity. The revised framing now asks whether temporal information generated by active sampling can help recover spatial details when images are spatially undersampled.
c. We clarified that the motion paradigm and parameter choices are biologically inspired, but not intended as a direct model of fixational eye movements or retinal circuitry. Results no longer state that the camera motion "mimics biological vision," but instead describes the controlled motion as a way to generate relative motion (which is needed to activate vent-based sensors) while avoiding display artifacts.
d. We edited the limitations around the Vernier experiment. The manuscript now states that the results provide a proof of feasibility for the temporal hypothesis, but cannot decisively resolve whether biological Vernier hyperacuity relies primarily on spatial or spatio-temporal information.
e. We added an explicit limitation that the biological comparison demonstrates only potential biological relevance, does not rule out alternative biological mechanisms, and requires further validation against human experiments and more biologically constrained models.

2. We discuss technological solutions that on the one hand followed key principles of biological systems but on the other hand were very simplified and only loosely reminiscent of their biological source. Those solutions eventually proved remarkably useful for gaining insights on the biological mechanism they were inspired by:

> "Engineering models inspired by biology have often become useful testbeds for neuroscience, even when the biological circuitry is not literally emulated. Convolutional neural networks, for example, have enabled systematic comparisons between task-optimized artificial representations and the primate ventral visual stream (e.g. Yamins et al. 2014), while recurrent neural networks trained on cognitive tasks have helped generate and test hypotheses about cortical population dynamics (e.g Mante & Sussillo 2013). We hope that the datasets introduced here will serve an analogous role for temporally precise, event-based representations. By isolating settings in which fine event timing matters, these datasets provide a controlled

testbed for studying spike-level codes, active sampling, and downstream decoding in artificial systems, and for comparing the resulting representations with neural or behavioral measurements in biological vision. Such comparisons can sharpen biologically motivated hypotheses without requiring the artificial models to be treated as mechanistic models of the brain."

Reviewer #3 (Remarks to the Author):

My main comment is that the manuscript is framed heavily around understanding mechanisms of biological hyperacuity, as it comes across from the introduction. The presented experiments are entirely computational, relying on event-camera data and transformer-based models. The work incorporates some biologically inspired design choices—such as simulated fixational eye movements, approximate matching of temporal and spatial scales. However, these elements primarily serve as parameter choices or arguments rather than direct empirical validation, and no direct link is established to neural processing or perception.

The main result—that precise temporal information is sufficient to decode hyperacuity-level spatial differences in an engineered vision system—is interesting and clearly demonstrated, and is consistent with known properties of biological vision such as the benefits of temporal integration and motion-induced sampling. However, it remains unclear what this implies for biological vision, where encoding mechanisms, noise characteristics, and downstream decoding constraints differ substantially. In particular, the emergence of hyperacuity-like behavior in a learning-based model operating on event-based data should not be taken as evidence that similar decoding strategies are used in the retina or early visual system.

As such, the work is best interpreted as a proof-of-principle demonstration of computational sufficiency under temporal encoding rather than as evidence for mechanisms underlying biological hyperacuity. Clarifying this distinction, or strengthening the connection to biological data or mechanistic models, would improve the manuscript. The paper is likely to be of strong interest to the computer vision and event-based sensing communities, but its relevance to biological vision is more limited, and the framing could be adjusted accordingly.

Reply:

We thank the Reviewer for voicing a concern about overestimating the relevance of our results for biological vision. We made a number of changes to reframe the contribution according to their request. Since this largely overlaps with comments of Reviewer #2. Please find the details in the reply to Reviewer #2.

Reviewer #4 (Remarks to the Author):

The authors have largely addressed my concerns and I am happy that they also found the suggested literature helpful and relevant.

I have one final comment related to the authors' reply "We thank the Reviewer for this important comment. We have now added these two relevant references to the Discussion (Lines 358-363):"

Line 302-307: The current phrasing implies that the mentioned references are exclusively about "higher-level perceptual inference or cortical learning" rather than related to "sensory encoding", which I think is not the case (e.g., learning is not addressed at all in these references).

Reference 55 is about visual spatial resolution and deals with mechanisms that are inherited from the retina. Additionally, to my understanding, reference 56 is much closer to the idea presented in the current study than the authors suggest with their current statement.

Specifically, Rekauzke et al. showed how a simultaneous counterchange in luminance is translated into asynchronous activation, thus, transforming a spatial event (i.e, luminance change at a stimulus border) into a temporal event. Their study suggests that temporal asymmetries may "influence perception of shape whenever abrupt shifts in visual objects or gaze cause counterchange of luminance at high-contrast borders".

This resonates very much with the idea of EB visual sensors that "… generate events asynchronously, when individual photosensors detect luminance changes [21], unlike frame-based (FB) cameras that collect synchronous information at fixed rates." (line 51-53).

Altogether, I think both references above should be more highlighted in the context of the ideas of the current study and at least ref. 56 should be mentioned also in the introduction.

Reply:

We thank the Reviewer for this helpful clarification.

We have revised the Introduction to make the connection to these sensory-encoding studies more explicit, particularly the relevance of Rekauzke et al. to temporally structured responses at luminance borders.

This places the prior biological work in context while preserving our distinct question: whether temporal information generated by active sampling can help recover spatial details when the image is undersampled in space.

> “Related sensory studies further show that visual spatial resolution can be shaped by mechanisms inherited from the retina (Kremkow et al. 2014), and that luminance counterchanges at high-contrast borders can be translated into asynchronous neural activation (Rekauzke et al. 2016). This transformation of a spatial discontinuity into a temporal signal parallels the operating principle of EB sensors, motivating a question relevant to both biological and artificial vision: can temporal information generated by active sampling help recover spatial details when the image is undersampled in space?”
